# A novel expression system for imaging single-molecule fluorescence in *Haloferax volcanii* WR806 enables visualization of altered Cas1 dynamics during UV-induced DNA damage response

**DOI:** 10.1093/femsml/uqag014

**Published:** 2026-04-13

**Authors:** Paula R Schrage, Uliana Afonina, Julia Wörtz, Anita Marchfelder, Koen J A Martens, James P Sáenz, Ulrike Endesfelder

**Affiliations:** Institute for Microbiology and Biotechnology, Rheinische Friedrich-Wilhelms-University of Bonn, 53115 Bonn, Germany; Institute for Microbiology and Biotechnology, Rheinische Friedrich-Wilhelms-University of Bonn, 53115 Bonn, Germany; B CUBE Center for Molecular Bioengineering, Technical University of Dresden, 01307 Dresden, Germany; Institute of Molecular Biology and Biotechnology of Prokaryotes, Ulm University, 89081 Ulm, Germany; Institute of Molecular Biology and Biotechnology of Prokaryotes, Ulm University, 89081 Ulm, Germany; Institute for Microbiology and Biotechnology, Rheinische Friedrich-Wilhelms-University of Bonn, 53115 Bonn, Germany; B CUBE Center for Molecular Bioengineering, Technical University of Dresden, 01307 Dresden, Germany; Faculty of Medicine, Technical University of Dresden, 01307 Dresden, Germany; Institute for Microbiology and Biotechnology, Rheinische Friedrich-Wilhelms-University of Bonn, 53115 Bonn, Germany

**Keywords:** single-molecule localization microscopy, single-particle tracking, *Haloferax volcanii*, cell biology, fluorescence microscopy, UV-light induced DNA damage, CRISPR-Cas Type I-B, FtsZ1

## Abstract

Fluorescence microscopy has become an indispensable tool in biological research, offering powerful approaches to study protein dynamics and cellular processes *in vivo*. Among archaea, *Haloferax volcanii* has emerged as a particularly well-suited model organism for imaging studies, with a growing toolkit of established fluorescent markers, plasmids, and promoter systems. Recent advances in single-molecule imaging techniques have created new opportunities through WR806, a carotenoid-free *H. volcanii* strain providing reduced autofluorescence background. However, existing plasmid-based expression systems in WR806 show critical limitations in protein expression control and challenges with protein aggregation. To address these limitations, we developed pUE001, a novel plasmid system specifically designed for WR806. This system achieves precise expression control by decoupling selection and induction through strategic implementation of the *trpA* selection marker. Through comprehensive characterization, we demonstrate that pUE001 provides superior control over protein expression compared to the previously established pTA962 system. It enables linear, titratable expression of diverse proteins—from the highly regulated CRISPR-Cas component Cas1 to the abundant structural protein FtsZ1—while preventing protein aggregation that could compromise native cellular functions. Additionally, we performed a comprehensive analysis of WR806 to show that carotenoid depletion does not affect native cellular physiology. Finally, to demonstrate the system’s utility, we investigated the role of Cas1 in UV-induced DNA repair using single-particle tracking photoactivated localization microscopy (sptPALM). Our findings reveal Cas1 colocalizing with DNA-dense cellular regions and significant, dose-dependent changes in Cas1 mobility following UV-light-induced damage, providing evidence for its possible involvement in DNA damage response processes and offering new insights into the expanding roles of CRISPR-Cas systems beyond adaptive immunity.

## Teaser

A robust single-molecule imaging approach in *Haloferax volcanii* enables tracking of Cas1 dynamics upon UV-induced DNA damage

## Introduction

Fluorescence microscopy has emerged as a powerful tool for cell biological research, enabling the study of protein dynamics, subcellular localization, and cellular biochemistry *in vivo*. Recent advances in tools for both diffraction-limited and super-resolution microscopy have established imaging as an essential method in archaeal research, providing direct visual insights into cellular processes (van Wolferen et al. 2022). For *Haloferax volcanii*, a model organism for halophilic archaea, there is a quickly growing toolkit available, e.g. fluorescent proteins (FPs) (Turkowyd et al. 2020, Ithurbide et al. 2024), plasmid systems (Allers et al. 2004, Ithurbide et al. 2024), and regulated promoters (Large et al. 2007, Rados et al. 2023), which has facilitated investigations into cell shape dynamics (Duggin et al. 2015, Walsh et al. 2019), DNA replication (Delpech et al. 2018), and cell division (Liao et al. 2021, Nußbaum et al. 2021, 2024).

As live-cell fluorescence microscopy becomes increasingly important to cell biology research, maintaining native cell biology while fluorescently labelling molecules of interest has emerged as a critical challenge. Two key issues have been reported for *H. volcanii*: First, genetic modifications, such as the deletion of essential genes for auxotrophic selection, can trigger unexpected metabolic effects, as demonstrated in this pleomorphic archaeon (Patro et al. 2023). Second, expressing genes from non-endogenous loci such as plasmid systems based on pHV2 (seemingly advantageous due to *H. volcanii’s* polyploid nature) can result in non-native transcription and protein production, potentially disrupting native biology through various mechanisms, including morphological variance (Patro et al. 2023), impaired cell division (Liao et al. 2021), altered protein dynamics, and the formation of protein aggregates such as inclusion bodies (Fahnert et al. 2004). Consequently, minimizing these artefacts requires thoughtful selection of strains, growth conditions, and expression systems.

Recently, our group established single-molecule localization microscopy (SMLM) and single-particle tracking (SPT) methods for *H. volcanii*. In this proof-of-concept work, we super-resolved FtsZ1 ring structures and tracked RNA polymerase dynamics in living *H. volcanii* cells (Turkowyd et al. 2020). A significant challenge arose from *H. volcanii*’s natural production of colorful carotenoids like bacterioruberin, generating high cellular autofluorescence and interfering with the detection of single fluorescent molecules in SMLM imaging. To overcome this limitation, we generated a carotenoid-free “albino” strain WR806 by disrupting the biosynthesis pathway through deletion of the *crtI* gene. Additionally, we established codon-optimized versions of two photoswitchable FPs using the tryptophan-inducible pTA962 plasmid expression system: PAmCherry1Hfx and Dendra2Hfx (Turkowyd et al. 2020).

Despite its advantages for single-molecule imaging, two critical issues emerged. First, although bacterioruberin is relatively scarce in *H. volcanii*, constituting less than 5% of cell membrane content (Kellermann et al. 2016), carotenoids in general have been shown to play a distinct role for different bacterial membranes, such as protection against oxidative stress (Kim et al. 2019, Rizk et al. 2021), alteration of membrane fluidity by increased production at lowered temperatures (Seel et al. 2020) or contributing to light scavenging in phototrophs (Polívka and Frank 2010). Even though WR806 exhibits wild-type-like growth (Turkowyd et al. 2020), the impact of carotenoid depletion on its membrane biophysics and consequent physiological effects in *H. volcanii* remained to be comprehensively investigated. Second, the plasmid system pTA962 presented significant challenges in controlling protein expression when used in WR806. For example, we found that protein aggregation and variable expression levels of proteins of interest (POIs) can compromise studies of native cell biology. In this work, this is best illustrated by the inclusion bodies formed during cultivation of WR806 pTA962-FtsZ1:Dendra2Hfx constructs, although this is a phenomenon that has also been widely reported in the literature (Prelich 2012, Cornet et al. 2023, Fujita et al. 2025). The use of plasmid-based systems for expressing modified proteins (e.g. fluorescently-labeled, mutated, or truncated proteins), is typically faster to experimentally implement when compared to genomic integration. While WR806’s multiple auxotrophic markers enable flexible selection strategies, they also create unique constraints for plasmid expression systems. Specifically, the strain carries deletions in *trpA* (tryptophan synthesis, tryptophan synthetase alpha subunit), *hdrB* (thymidine synthesis), *leuB* (leucine synthesis), and *pyrE2* (uracil synthesis) genes (Ortenberg et al. 2000, Bitan-Banin et al. 2003, Allers et al. 2004). While these deletions enable experiments based on multiple plasmids, they necessitate—if not complemented by plasmids carrying the corresponding synthesis genes—external supplementation of tryptophan, thymidine, and uracil (leucine supplementation can be typically neglected as it is present in standard growth media). Consequently, effective plasmid systems for WR806 must satisfy two key criteria: compatibility with this auxotrophic background and controlled protein expression through inducible promoters to minimize disruption of native cell biology.

Currently, *H. volcanii* has only two available inducible promoter systems: p.tna, the native promotor of the tryptophanase gene *tnaA* (HVO_0009) (Large et al. 2007) and p.xyl-750, the native promoter of the xylose degradation operon *xacEA* (HVO_B0027-28) (Rados et al. 2023). Due to high leaky expression observed with p.xyl-750 (Rados et al. 2023), we focused on the rather tightly controlled tryptophan-inducible p.tna system (Large et al. 2007). However, the previously used pTA962 plasmid (Turkowyd et al. 2020), presents a significant limitation when used in WR806: while pTA962 complements *hdrB* and *pyrE2* selection markers in WR806, it lacks *trpA* complementation needed for tryptophan synthesis. This combination of selection markers renders WR806 carrying pTA962 dependent on external tryptophan supply in the medium. The direct tryptophan uptake from the medium creates an inherent problem as the plasmid’s tryptophan-inducible p.tna promoter [originally designed for strains with genomic *trpA* for internal synthesis, (Allers et al. 2010)] is induced even at the minimal tryptophan concentrations required for growth. This unintended coupling of selection and induction leads to constitutive POI expression from pTA962. Two strategies can address this issue: 1) engineering a *crtI*-gene-deficient mutant of a *trpA*-encoding strain like H26 or H98 (Allers et al. 2004), or 2) incorporating *trpA* directly onto the plasmid used for POI expression. We preferred the latter approach, since it maintains minimal use of selection markers.

The native promoter of the *trpCBA* operon is not tightly regulated (Large et al. 2007). At the same time, expression levels of POIs under a p.tna promoter are tightly regulated in strains carrying genomic *trpCBA* (Large et al. 2007). We thus hypothesized that providing a plasmid-based copy of *trpA* under the constitutive p.fdx promoter would result in a similar, post-transcriptional, control of tryptophan synthesis as for the native *trpCBA* operon. To test this, we developed pUE001, a novel expression plasmid that combines the backbone from pTA231 [encoding the *trpA* selection marker under the control of p.fdx, (Allers et al. 2004)], with the p.tna promoter region from pTA962 (Allers et al. 2010). Here we show that this system effectively decouples induction from selection, providing improved control over protein expression levels. Furthermore, by design, pUE001 utilizes only the *trpA* selection marker, flexibly preserving WR806’s remaining auxotrophic markers (*hdrB, leuB*, and *pyrE2*) for additional genetic manipulations.

We characterize the performance of this new plasmid system in three cases: First, by expressing cytosolic Dendra2Hfx alone, and then by testing two proteins representing two extremes of protein abundance and stability. The second protein we chose is the highly abundant and stable cytoskeletal protein FtsZ1, while the third protein Cas1, is a highly regulated, low-abundant protein of the type I-B Clustered Regularly Interspaced Short Palindromic Repeats and CRISPR-associated genes (CRISPR-Cas) system in *H. volcanii*. Furthermore, for Cas1, we anticipated that if pUE001 enables reliable expression in WR806, thereby yielding a robust low-expression regime within the native range of Cas1 in individual cells, it would allow us to visualize and quantify its transient interaction dynamics following UV-induced damage using *in vivo* single-molecule imaging. This data derived from single-particle tracking photoactivated localization microscopy (sptPALM) *in vivo* would potentially add further evidence for the reported role of Cas1 in DNA repair as established by previous studies employing mutant and complementation growth assays, pull-down analyses, and processing of flapped and branched DNA templates (Babu et al. 2011, Rollie et al. 2015, Wörtz et al. 2022).

To summarize, this work makes three key contributions: First, we present a detailed comparative analysis of WR806 demonstrating that carotenoid depletion does not affect native cellular processes, particularly membrane biophysics. Second, we establish that our novel plasmid pUE001, combined with the carotenoid-free strain WR806, creates an ideal tool for low-background fluorescence imaging in *H. volcanii*. Finally, we validate its applicability by successfully tracking the single-molecule dynamics of Cas1—which was unachievable with previous expression systems—and, provide first single-molecule imaging evidence of Cas1 being involved in the cellular DNA repair response upon UV damage.

## Material and methods

### Strains, media, and cultivation conditions

The strains used in this study are listed in Supplementary Table S1.

*Escherichia coli* strains were grown over night at 37°C in LB liquid culture shaking at 180 rpm or on LB Agar plates with 1.5% (w/v) agar–agar. Agar plates and liquid cultures were supplied with a final concentration of 100 µg/ml of carbenicillin for selection of plasmids.

All *H. volcanii* strains were grown at 42°C in liquid HV-Cab medium (Duggin et al. 2015, De Silva et al. 2021) or HV-YPC medium. HV-Cab agar plates with 1.5% (w/v) agar–agar were used to streak out strains from cryostocks or for plating after transformation. The additives uracil, thymidine and tryptophan were added in final concentrations of 0.45 mM, 0.16 mM, and 0.25 mM, respectively. WR806 and H119 were supplied with additional tryptophan, thymidine and uracil (Supplementary Table S1). WR806 carrying pTA962 plasmid constructs were supplied with tryptophan, while pUE001 carrying strains were supplied with thymidine and uracil (Supplementary Table S1). Induction of the p.tna promoter system was accomplished by supplying strains with tryptophan in concentrations between 0.25 and 5 mM.

### Determination of growth rates

Growth rates of H119 and WR806 were measured using a Tecan Infinite® M PLEX plate reader. Single colonies were selected from agar plates, and cultures were inoculated in either HV-Cab medium supplemented with 0.45 mM uracil, 0.16 mM thymidine, and 0.25 mM tryptophan or HV-YPC medium supplemented with 0.16 mM thymidine and grown to stationary phase. Cultures were then diluted to an OD_600_ of 0.1 and transferred into a 48-well plate. Growth curves were monitored over 72 h at 42°C, with brief shaking performed every 20 min before OD measurements were taken.

Growth experiments of UV-treated WR806 pUE001-Cas1:Dendra2Hfx were performed in HV-CAB medium supplied with 0.45 mM uracil and 0.16 mM thymidine as described above. Immediately before transfer into the plate reader, cells were diluted to an OD of 0.1 and 2 ml of culture was centrifuged into a well of a 48 well plate at 2000 rpm and 25°C for 10 min, by two consecutive 1 ml centrifugation steps. Prior to the UV treatment 800 µl of the supernatant were removed and the cells were treated with a Thorlabs M265L5 UV lamp (265 nm, 38.4 mW, 440 mA). The samples were exposed to the UV light for 235 ms (50 J/m^2^), 470 ms (100 J/m^2^), and 940 ms (200 J/m^2^). Subsequently, the wells were filled back up with fresh HV-Cab medium containing uracil and thymidine in the abovementioned concentrations before starting the measurement.

### Construction of plasmids

Vector constructs were created by Gibson assembly using the NEBuilder® HiFi DNA Assembly Cloning Kit (NEB, Frankfurt am Main, Germany) according to the manufacturer’s protocol. All oligonucleotides to amplify backbones and inserts are listed in Supplementary Table S2, all plasmids are listed in Supplementary Table S3. Backbones and inserts were amplified by PCR with Q5® High Fidelity DNA Polymerase (NEB, Frankfurt am Main, Germany). To construct the plasmids pUE001-FtsZ1:Dendra2Hfx (Addgene # 234 671) and pUE001-Cas1:Dendra2Hfx (Addgene # 234 670), the plasmid pTA231-p.Syn-Dendra2Hfx was used as the template for backbone amplification. To amplify the promoter and terminator region, including the coding sequence for the fusion proteins FtsZ1:Dendra2Hfx and Cas1:Dendra2Hfx, the plasmids pTA962-FtsZ1:Dendra2Hfx and pTA962-Cas1:Dendra2Hfx were used as templates. Consecutively, pUE001-Cas1:Dendra2Hfx was used to construct pUE001-Dendra2Hfx (Supplementary Fig. S1, Addgene # 234 669). To reduce the number of original template plasmids prior to transformation, all products were digested using DpnI (NEB, Frankfurt am Main, Germany).

### Strain creation

Vector constructs were transformed into competent *E. coli* DH5α cells (NEB, Frankfurt am Main, Germany) via heat-shock to ensure efficient plasmid replication. Plasmid sequences were verified using Sanger and Oxford Nanopore sequencing (Eurofins genomics, Cologne, Germany). The plasmids were then passaged through *E. coli dam-/dcm-* (NEB, Frankfurt am Main, Germany) to obtain non-methylated plasmid DNA, which was subsequently transformed into *H. volcanii* WR806 using PEG600 (Cline et al. 1989). Transformed cells were streaked onto HV-Cab agar plates containing the respective selection additives (Supplementary Table S3) Positive transformants were checked by colony PCR (Supplementary Table S2).

### Lipid extraction

**Figure 1:**
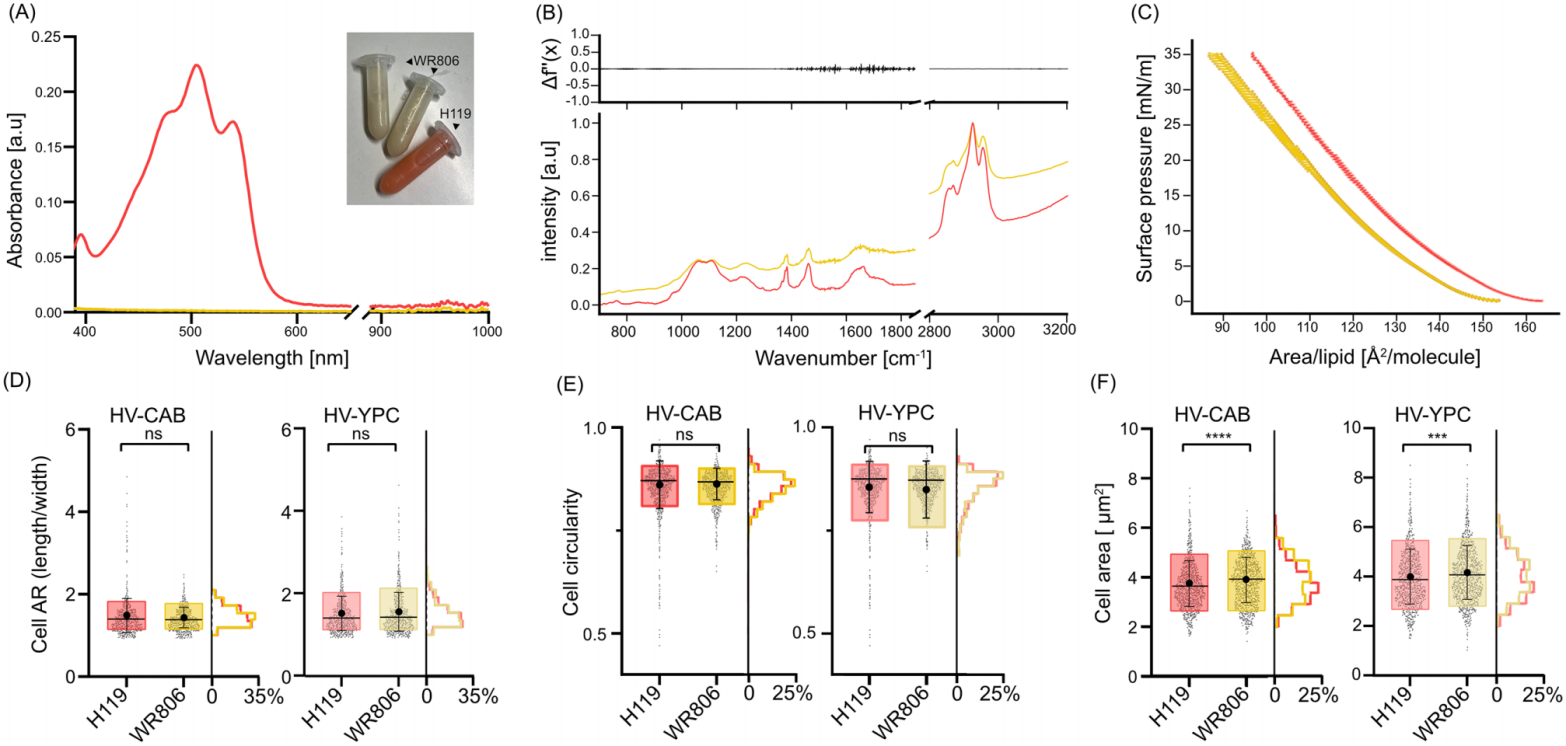
Comparative characterization of *H. volcanii* strains H119 and WR806. Strain H119 [DS70-wildtype (ΔpHV2), *∆pyrE2, ∆trpA, ∆leuB*; (Allers et al. 2004)] was compared to the carotenoid-deficient “albino” strain WR806 [DS70-wildtype (ΔpHV2), *∆pyrE2, ∆trpA, ∆leuB, ∆hdrB, ∆crtI*; (Turkowyd et al. 2020)], with *∆crtI* deletion being responsible to disrupting carotenoid biosynthesis. Data from H119 and WR806 are shown in red (left samples in lower panels) and yellow (right samples in lower panel), respectively. (A) UV-VIS absorbance spectra (technical duplicates) with inset showing Eppendorf tubes containing total lipid extracts. (B) FTIR spectra, normalized by intensity, of lipid extracts (technical duplicates; lower panel) and their second-order derivative differences (upper panel). (C) Area isotherms measuring surface pressure versus molecular area for lipid monolayers at 42°C (two biological replicates, each with five technical replicates). (D–F) Quantitative morphological analysis of cell populations by microscopy, showing distributions of (D) aspect ratio, (E) circularity, and (F) cell area per single cell (n > 950 cells per strain). Box plots display 10%–90% quartiles with mean (big circle), median (line) and standard deviation. Histograms show frequency distributions. Significance by Mann–Whitney-test. ns: not significant; ****: *P* < 0.0001; ***: *P* = 0.0002.

Lipid extracts used for absorbance spectra, FTIR spectra and monolayer assays (Fig. 1 and Supplementary Fig. S2) were obtained from H119 and WR806 grown from single colonies in HV-Cab medium to stationary phase. Lipid extraction was performed following a standard protocol (Bligh and Dyer 1959) with modifications. Briefly, cells were pelleted at 6000 rpm for 15 min and separated into 25 mg pellets. Pellets were washed with PBS and cell lysis was achieved by sonication for 20 min. Subsequently, 300 µl chloroform: methanol (1:1, v/v), 100 µl 0.1 M HCl, and 100 µl 0.5 M NaOH were added. Samples were shaken vigorously for 5 min, followed by 40 min centrifugation at 9000 rpm. Again, 200 µl of 0.1 M HCL and 0.5 M NaOH were added, respectively, and samples were shaken vigorously for 15 min. Lastly, after centrifugation for 3 min at 13 000 rpm, the organic, lower phase was removed from the sample and solvents were evaporated under nitrogen flow. Lipids were stored at −20°C until further analysis.

### Phospholipid quantification

The concentration of phospholipids was quantified as described in (Katewa and Katyare 2003). Briefly, phosphorus ICP standard solution (Merck, Germany) at concentrations of 0, 5, 10, 50, and 100 nmol was used to determine the standard curve. Samples and standards were mixed with 500 µl 70% HClO_4_, mixed thoroughly and incubated at 200°C for 2 h. After 30 min of cooling, 500 µl 10% (w/v) ascorbic acid (stock solution in dH2O) and 500 µl 2.5% (w/v) (NH_4_)_6_Mo_7_O_24_ · 4 H_2_O (stock solution in dH2O) was added to each sample, mixing in between steps. After 30 min incubation at 37°C, absorbance of each sample was measured at 820 nm wavelength in a plate reader. Concentrations of lipid extracts were calculated based on the standard curve, which was plotted as linear fit of the A_820_ function of moles of inorganic phosphate.

### Lipid monolayer assay

Lipid monolayer assays were performed at 37°C and 42°C using a Langmuir Blodgett trough Microtrough G1 (Kibron, Finland) and using the lipid extracts from H119 and WR806 cultures. Chloroform solutions of lipid extracts were directly prepared at the concentrations determined by the phosphate assay. Monolayers were formed by injecting 13 µl and 30 µl of lipid solution onto an aqueous subphase (buffer of 3.3 mM sodium citrate, 3.3 mM sodium phosphate, 3.3 mM glycine, and 0.15 M NaCl at pH 7.5) maintained at 37°C and 42°C, respectively, using a built-in temperature-controlled circulating water bath. Isotherms were recorded with a 70 cm^2^ Teflon Langmuir trough, which was equipped with a motorized compression barrier and a pressure sensor (Kibron DeltaPi, Finland). The area per lipid was calculated by dividing the total lipid area by the number of lipid molecules as determined by a phosphate quantification assay (Katewa and Katyare 2003). For each mixture, the area per lipid was estimated from the averages of isotherms obtained from two monolayers, with three technical replicates for 37°C and five replicates for 42°C. The areas corresponding to the closest pressure values to the defined pressure points (5, 10, 15, 20, 25, 30, and 35 mN/m) were selected for the averaged isotherms. The compressibility modulus (k) (Supplementary Fig. S2B, C) represents the membrane’s resistance to compression and was also calculated using the Langmuir monolayer experimental data. Compressibility is defined as: k = -A * (∂π/∂A)T where A is the molecular area, π is the surface pressure, and the subscript T indicates the derivative is taken at constant temperature (Przykaza et al. 2019). This dimensionless parameter quantifies how the molecular area changes in response to applied surface pressure.

### FTIR-spectroscopy

For FTIR spectroscopy measurements of lipid extracts from H119 and WR806, the samples were embedded into KBr optical windows. First, 50 µl sample was mixed with 250 mg of finely ground, dry KBr powder. The dried sample was pressed under 8T pressure for 5 min. FTIR spectra were recorded on an INVENIO Tensor spectrometer (Bruker, USA), with a KBr window used as the background. All spectra were processed and analyzed using the OPUS software (Bruker, USA), with normalization and peak searching performed by a custom Python code.

### Plate reader experiments

**Figure 2:**
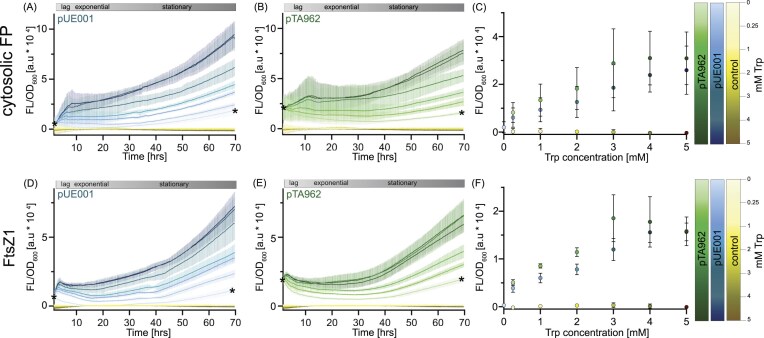
Fluorescence characterization of Dendra2Hfx expression in *H. volcanii* strain WR806. (A, B, D, E) Time-course fluorescence measurements of tryptophan-induced protein expression normalized to cell density OD_600_ (ex 470 nm; em 501–519 nm, optimized for Dendra2Hfx green fluorescence). Measurements were performed at 42°C in biological triplicates, each with technical duplicates, data is visualized as mean with standard deviation. (A, B) Cytosolic Dendra2Hfx expression from pTA962 (green, right panel) and pUE001 (blue, left panel) plasmids (plasmid details in Supplementary Table S3; Supplementary Fig. S1). (D, E) FtsZ1:Dendra2Hfx fusion protein expression from the same plasmid systems. Cultures were induced with tryptophan concentrations of 0, 0.25, 1, 2, 3, 4, and 5 mM. Plasmid-free WR806 (yellow) served as a negative control. Initial fluorescence reflects protein accumulation from autoinduction of three-day stationary phase pre-cultures. Asterisks at 0 and 70 h indicate the matching fluorescence levels of initial values and stationary phase cultures. Growth phases (lag, exponential, and stationary) were determined from raw data of OD_600_ measurements (Supplementary Fig. S3). (C, F) Steady-state fluorescence levels during exponential growth phase, with cell growth, protein production and protein degradation rates approaching an equilibrium.

Plate reader experiments on a Tecan Infinite® M PLEX were performed at 42°C for time-course measurements of OD_600_ and fluorescence intensity and in dependency of tryptophan concentration used for induction of the p.tna promoter. Single colonies were picked from HV-Cab agar plates and inoculated in selective HV-Cab medium until the stationary phase was reached. Prior to the plate reader run, cultures were diluted to OD_600_ 0.1 with final tryptophan concentrations between 0 and 5 mM. All samples were measured in biological triplicates with technical duplicates each in Greiner µClear® CELLSTAR® black 96 well plates. Measurements were taken every 20 min after a brief orbital shaking step of 4 mm for 30 s. OD_600_ measurements were blanked using the medium values from the respective runs. Fluorescence was measured at an excitation wavelength of 470 nm and emission window of 510 ± 9 nm matching green Dendra2Hfx fluorescence. The gain was determined for each POI individually. Fluorescence measurements were blanked to the average fluorescence of WR806 WT as a negative control over time. To obtain the normalized fluorescence intensity curves of Fig. 2, measured fluorescence intensities were divided by the respective OD measurement, to normalize to the cell density.

### In-gel fluorescence SDS-PAGE

Whole cell lysates were obtained from WR806 wildtype, WR806 pUE001-Cas1:Dendra2Hfx, and WR806 pUE001-FtsZ1:Dendra2Hfx to determine fusion protein size via in-gel fluorescence SDS-PAGE. Briefly, precultures of the respective strain were inoculated from single colonies picked from agar plates and grown to stationary phase. 250 ml main cultures were inoculated with highly induced conditions (4 mM) over night at 42°C and 180 rpm shaking. Subsequently, cultures were centrifuged in 2 × 50 ml Aliquots at 4000 rpm and 4°C for 30 min and washed with enriched PBS (2.5 M NaCl, 0.15 M MgCl_2_, 10% 10 x PBS). After washing, the samples were centrifuged with the same parameters described before and the pellets were resuspended in 4 ml Lysis buffer [100 mM Tris-HCl (pH 7.5), 10 mM EDTA]. Cells were lysed by ultrasonication and the cell debris was separated from the soluble fraction by centrifuging at 4000 rpm for 45 min at 4°C. The protein concentration of the respective cell lysates was determined by BCA assay (Thermofisher, Germany).

To determine the protein size, cell lysates and as a control purified Dendra2 from *E.coli* (Turkowyd et al. 2017) were mixed with 4 × SDS loading buffer [125 mM Tris-HCl (pH 6.8), 50% glycerol, 4% SDS, 0.02% bromphenol blue, 10% β-mercaptoethanol] and incubated at 30°C for 10 min to cover protein surface charges while maintaining fluorescence of the FP based on a previously described protocol (Sanial et al. 2025). Protein concentrations were adjusted to compensate for the low-abundance of Cas1 (20 µg/lane WR806 wildtype, 20 µg/lane WR806 pUE001-FtsZ1:Dendra2Hfx, 40 µg/lane WR806 pUE001-Cas1:Dendra2Hfx). The samples were then separated using a standard discontinuous SDS-PAGE with a 12.5% separation gel. PageRuler Plus Prestained Protein Ladder (Thermofisher, Germany) was used as a molecular weight maker. The SDS-PAGE was briefly washed with dH2O and bands were visualized using the Typhoon™ Amersham™ Biomolecular Imager (Cytiva, USA). To visualize Dendra2, the fusion proteins Cas1:Dendra2Hfx, FtsZ1:Dendra2Hfx, and the pre-stained molecular weight marker, the Cy2, Cy3, and Cy5 channel were imaged consecutively with a pixel size of 50 µm. Optimal PMTs were determined by a pre-run. After visualization the images were overlaid in Fiji 1.54f (Schindelin et al. 2012).

### Detection of UV-induced DNA damage by Endonuclease V treatment and denaturing agarose gel electrophoresis

To quantify dosage dependent UV-induced DNA damage from live cell cultures a T4 Endonuclease V based assay was used. T4 Endonuclease V (T4 PDG) digests cyclobutene pyrimidine dimers (CPDs), which are one of the most common forms of UV-induced DNA damage. To perform the assay, WR806 pUE001-Dendra2Hfx was inoculated in HV-Cab with 0.45 mM thymidine and 0.16 mM uracil to an OD of 0.3. Subsequently, 1 ml of culture per well was centrifuged in a 48 well plate at 2000 rpm and 25°C for 10 min to collect cells at the bottom of the well. After centrifugation, 800 µl of medium were removed from each well and the samples were treated with a Thorlabs M265L5 UV lamp (265 nm, 38.4 mW, 440 mA). Cells were exposed to the UV light for 470 ms (100 J/m^2^), 940 ms (200 J/m^2^), 1880 ms (400 J/m^2^), 3760 ms (800 J/m^2^), and 7520 ms (1600 J/m^2^), respectively. After the UV-treatment, eight wells per condition were pooled and plasmid DNA was purified using the Monarch® Plasmid Miniprep Kit (New England Biolabs, Frankfurt am Main, Germany) according to the manufacturers protocol. As DNA quantification via nanodrop measurements could not be performed in a robust fashion, isolated plasmid DNA for each condition was split into two and either treated with Endonuclease V or left untreated, for relative quantification. Briefly, 20 µl of plasmid DNA were mixed with 5 µl rCutsmart buffer, 1 µl T4 Endonuclease V (T4 PDG), 23 µl dH_2_O, and 1 µl BamHI-HF to linearize the plasmid (all reagents from New England Biolabs, Frankfurt am Main, Germany). The untreated control samples were mixed with 1 µl of dH_2_O instead of T4 Endonuclease V. The samples were inoculated at 37°C for 30 min. As T4 Endonuclease V introduces single-strand nicks it is necessary to denature the DNA to successfully visualize decreasing band sizes. To achieve denaturing conditions the samples were mixed with urea loading dye (8 M urea, glycerol 30%, 3.7 mM bromphenole blue, 6.2 mM crescol red, 2% SDS, dissolved in 1 x TAE) and incubated at 98°C for 30 min, based on a previously described protocol (Hegedüs et al. 2009). Additionally, 5 µl 1 kb purple ladder (New England Biolabs, Frankfurt am Main, Germany) was mixed with 2 µl urea loading dye and incubated at the same conditions as the samples to be used as a denaturing control. Sample, denatured ladder and non-denatured ladder were loaded onto a 1% Agarose gel containing 1 M Urea. The gels were run in 1 X TAE buffer with 1 M Urea at 100 V for 1 h and visualized using a ChemiDoc Imaging System (Bio-Rad, Germany). Band intensities were quantified using the ROI manager and measurement feature in Fiji 1.54f (Schindelin et al. 2012). An exponential decay was fitted to the mean intensity values using GraphPad Prism 10.4.1 (GraphPad Software, LLC).

### Microscopy

All imaging was performed using an epifluorescence microscope custom-built on a Ti Eclipse body with PFS focus stabilization system (Nikon, Düsseldorf, Germany), built upon and modified from a previous version of the system as described in (Martens et al. 2024). The setup is equipped with five laser lines covering 405 nm, 488 nm, 638 nm, 750 nm (Oxxius, Lannion, France), and 561 nm (Novanta, Boston, USA) wavelengths which are coupled into a 70 μm diameter multi-mode fibre (CeramOptec, Bonn, Germany) by a protected silver reflective collimator (RC04FC-P01, Thorlabs), and then expanded and focused by several optical elements and a dichroic ZT405/488/561rpc (Chroma, Bellows Falls, VT, USA) onto the back focal plane of a CFI Apo TIRF 60 x oil objective (NA 1.49, Nikon). To ensure uniform illumination and eliminate speckle patterns, the fiber is mechanically vibrated using a small eccentric mass motor. The heating stage was set to 37°C for all imaging experiments. Fluorescence was recorded by a Prime BSI sCMOS camera (Teledyne Photometrics, Tucson, AZ, USA; 107 nm pixel size) and using ZET405/488/561m-TRF (Chroma) and bandpass filters ET510/80 m (green Dendra2Hfx), ET610/75 m (orange Dendra2Hfx) and 460/50 nm emission filter (Nikon, Düsseldorf, Germany). The microscope setup was controlled by μManager 2.0 (Edelstein et al. 2014) combined with Pycro-Manager 1.0.0 (Pinkard et al. 2021), while laser triggering was managed by a TriggerScope 4 (Advanced Research Consulting, Newcastle, CA, USA).

### Agarose pads

Agarose pads were prepared with 1.5 (w/v%) low-melting (Sigma–Aldrich, Germany) agarose and the respective HV-Cab medium contained the required selection additives. Microscopy slides and coverslips were cleaned in 1 M KOH overnight. The agarose was melted at 70°C for 20 min and cooled to 42°C once fully melted. The melted agarose was then spotted onto dimpled microscopy slides (Thermofisher, Germany) and covered with coverslips to solidify.

For sptPALM imaging, plane microscopy slides and coverslips were subsequently cleaned with detergent, dH_2_O, 70% Ethanol and 1 M KOH. Gene Frame stickers (Thermofisher, Germany) were used to spot the samples onto the microscopy slides and then covered by cover slips.

### Cell morphology imaging and analysis

To analyze H119 and WR806 cell morphology, the respective strains were inoculated in HV-Cab or HV-YPC medium to stationary phase until an OD_600_ of 2 was reached. All cultures were then sub-cultured to an OD of 0.05 and grown until they reached OD 0.1. Cultures were prepared for imaging by centrifuging 2 ml of culture at 4000 rpm for 5 min and washed with the respective medium. Cell pellets were then resuspended in medium and spotted onto agarose pads. Brightfield images were obtained by acquisitions of brightfield movies (500 frames, 50 ms) and averaging the images using Fiji 1.54f (Schindelin et al. 2012). Cells were segmented using StarDist 0.3.0 (Schmidt et al. 2018, Weigert et al. 2020) with a pretrained model for *H. volcanii*, and cell shape descriptors [length, width, area, aspect ratio (length/width), circularity (4π × area/perimeter²)] were measured. Wrongly segmented cells were manually corrected. For significance testing, a Mann–Whitney-U test was performed.

### Diffraction-limited fluorescence imaging and data analysis

Cell samples were grown to stationary phase in HV-Cab medium prior to imaging and sub-cultured with the specified conditions into fresh HV-Cab with 0, 0.25, or 4 mM tryptophan and grown to the stated time points. Prior to imaging, cells were harvested in a microcentrifuge at 4000 rpm for 5 min and washed two times with fresh medium. Cells were then resuspended in a smaller volume of fresh medium and spotted onto agarose pads. Prior to fluorescence read-out, brightfield images were obtained to serve as templates for cell segmentation. Green Dendra2Hfx (Turkowyd et al. 2020) was excited with a 488 nm laser at a laser power of 2 W/cm^2^ for 500 frames with 50 ms exposure. To obtain average fluorescence intensities per cell area, images were analyzed using a custom Python script. First, brightfield images were averaged and segmented with StarDist 0.8.5 (Schmidt et al. 2018, Weigert et al. 2020) with a pre-trained model for *H. volcanii*. Cells that were not properly segmented or cut-off by an image edge were manually excluded. The average fluorescence intensity per cell was calculated from averaged fluorescence images, normalized to the background intensity. To quantify the number of cells carrying fluorescence spots, cells were counted manually.

### Inducing DNA damage by UV light exposure of cell cultures for sptPALM imaging

WR806 pUE001-Cas1:Dendra2Hfx was grown from a single colony to stationary phase in HV-Cab medium supplied with 0.16 mM thymidine and 0.45 mM uracil (without tryptophan). Prior to imaging, the culture was sub-cultured to OD_600_ 0.02 with a final concentration of 0.25 mM tryptophan and grown overnight under inducing conditions. The next morning, the culture was diluted to an OD_600_ of 0.1. To expose the cells to UV-light, 3 ml of the culture were centrifuged in a 48-well plate, consecutively. Before UV light exposure using a Thorlabs M265L5 UV lamp (265 nm, 38.4 mW, 440 mA), 800 µl of the supernatant was removed to prevent UV absorption by the medium. Cells were exposed to the UV light for 235 ms (50 J/m^2^), 470 ms (100 J/m^2^), and 940 ms (200 J/m^2^), respectively. Afterwards, cells were quickly resuspended in 1 ml HV-Cab medium with 0.45 mM uracil, 0.16 mM thymidine and 0.25 mM tryptophan and grown for varying recovery times at 42°C and 180 rpm. Before imaging, cells were centrifuged at 4000 rpm for 5 min and resuspended in a smaller volume of medium, before spotting onto agarose pads. Cell samples with the shortest recovery time of 15 min were spotted directly onto prepared agarose pads.

### sptPALM imaging and analysis

First, brightfield images (200 frames, 50 ms exposure) were obtained to record cell outlines. To track single Cas1:Dendra2Hfx fusion proteins, primed photoconversion to photoconvert Dendra2Hfx using a 488 nm and 750 nm laser was used (Turkowyd et al. 2017). At least 10 000 frames with an exposure time of 20 ms each were recorded for each field of view. To control FP activation, the 488 nm laser was strobed for 1 ms at the beginning of each frame and laser power was increased continuously during the acquisition. Converted Dendra2Hfx was recorded by the 561 nm laser, strobed for 10 ms in the middle of each frame to avoid motion blur. The first 1000 frames of each acquisition were exposed only to the 561 nm laser to bleach noise and discarded from analysis.

Localization finding and single-particle tracking was performed with constant parameters for all datasets to ensure comparability (Supplementary Table S4, S5). Localization of emitters was performed using ThunderSTORM 1.3 (Ovesný et al. 2014). Tracking was performed by custom software (*swift* v0.4.3, Endesfelder lab, beta-test software available upon request to the authors) and mean jump distances (MJD) and motion types were analyzed. Weighted MJD (wMJD) data sets were subsampled by removing 40% of data using a custom python script. A Mann–Whitney-U test was performed on each subsampled data set to investigate differences between conditions. The untreated control data set was randomly split into two parts to serve as a control condition. U-values were normalized to the respective sample size of both compared groups. The mean, the median and the 5% and 95% confidence intervals were calculated based on the normalized U-value distributions. If U-value distributions were truncated at 0.5 a Gaussian fit was applied to the truncated frequency distributions and statistical parameters were determined based on the fitted curve.

### Deconvolution

To deconvolute DNA in WR806 pUE001-Cas1:Dendra2Hfx, cultures were grown and induced as described above and were mixed with Hoechst 33 342 (Life Technologies, Thermofisher, Germany) at a final concentration of 5 µg/ml and samples were incubated at 42°C with shaking for 10 min prior to imaging. Subsequently, the samples were prepared as described above for sptPALM imaging. After recording sptPALM of Cas1:Dendra2Hfx, Hoechst 33 342 was visualized using a 405 nm laser for excitation recording z-stacks with a 14 nm step size. All images were deconvolved with Huygens Professional version 25.04 (Scientific Volume Imaging, The Netherlands, http://svi.nl), using the CMLE algorithm. Acuity, SNR, iterations and background values were adjusted to the respective sample. To overlay localizations and deconvolved images the maximum intensity projection (MIP) was used. Deconvolved images were sized up 10-fold using a bicubic interpolation algorithm to match super-resolved images of Cas1:Dendra2Hfx localizations.

Cas1:Dendra2Hfx was tracked and filtered as described above and localizations (JD under 250 nm, JD between 250 nm and 500 nm, JD under 500 nm) were exported from *swift* v0.4.3 (Endesfelder lab). Localizations were visualized using Thunderstorm (Ovesný et al. 2014) and blurred according to their localization precision (Endesfelder et al. 2014) to approximate a density map of Cas1:Dendra2Hfx and match the deconvolved image. Deconvolved DNA and localization images were aligned manually using a line ROI in Fiji (Schindelin et al. 2012).

### Software

Custom scripts were written with Python 3.11.5 using pandas, matplotlib, numpy, skimage, csbdeep, stardist, seaborn, and tiff-file libraries. GraphPad Prism 10.4.1 (GraphPad Software, LLC) was used for plotting and statistical tests. Affinity Designer 1 (Serif, Ltd) was used to finalize figures. *swift* v0.4.3 (Endesfelder lab, beta-test software available upon request to the authors), Pycro-Manager 1.0.0 (Pinkard et al. 2021) and Fiji 1.54f (Schindelin et al. 2012) with plugins for µManger 2.0 (Edelstein et al. 2014), Stardist 0.3.0 (Schmidt et al. 2018, Weigert et al. 2020), and Thunderstorm 1.3 (Ovesný et al. 2014) were used for image analysis and tracking. FTIR spectra were analyzed using OPUS (Bruker, USA). with Huygens Professional version 25.04 (Scientific Volume Imaging, The Netherlands, http://svi.nl) was used to deconvolve images.

## Results and discussion

### Characterization of the carotenoid-deficient WR806 strain

Natural carotenoid synthesis of *H. volcanii* is facilitated by the phytoene dehydrogenase encoded by *crtI* (HVO_2528). The produced carotenoids cause significant cellular autofluorescence under fluorescence excitation, a major caveat for SMLM imaging as it hinders the detection of single molecules (Turkowyd et al. 2020). To overcome this limitation, the “albino” strain WR806 [DS70-wildtype (ΔpHV2), *∆pyrE2, ∆trpA, ∆leuB, ∆hdrB, ∆crtI*], with disrupted carotenoid biosynthesis by a *∆crtI* deletion was created (Turkowyd et al. 2020). Given the unresolved open question of whether carotenoid depletion might affect native cellular processes, particularly membrane biophysics, we now performed detailed comparative analyses between WR806 and the red carotenoid-producing strain H119 [DS70-wildtype (ΔpHV2), *∆pyrE2, ∆trpA, ∆leuB*; (Allers et al. 2004)]. Analysis of lipid extracts from H119 and WR806 cultures via visual inspection and absorption spectroscopy confirmed absence of carotenoids in WR806 extracts, and characteristic absorption of carotenoids in the blue to green spectrum between 410 nm and 590 nm (Fig. 1A). To characterize the molecular composition of these extracts, we performed Fourier-transformed infrared (FTIR) spectroscopy. The FTIR spectra, normalized to intensity of the asymmetric CH_2_ vibration peak at 2925 cm^−1^ associated with the lipid side chains (Movasaghi et al. 2008), demonstrated remarkable compositional similarity between WR806 and H119 lipid extracts (Fig. 1B). While absolute peak intensities varied due to different lipid concentrations, second derivative analysis confirmed the nearly identical chemical composition of both extracts. Notably, the characteristic bacterioruberin peak at 1650 cm^−1^ (Noby et al. 2023) showed only minimal variation between samples.

We assessed the biophysical membrane properties of the lipid extracts using Langmuir-Blodgett monolayer compression assays under two conditions: physiological (pH 7.5, 42°C; Fig. 1C) and SMLM imaging conditions (pH 7.5, 37°C; Supplementary Fig. S2A). The surface-pressure-area isotherms, normalized to lipid molecule numbers quantified by a phosphate assay, were analyzed for changes in compressibility—a sensitive indicator of phase transitions and alterations in lipid ordering (Risović et al. 2011). Identical slopes were observed under both conditions, with no inflection points and thus no evidence of phase transitions, suggested equivalent biophysical behavior between the extracts. The observed lateral displacement of isotherms along the x-axis can be attributed to quantification limitations of the phosphate assay, which fails to detect non-phosphate-containing membrane components such as sterols, quinones, and carotenoids. Given that *H. volcanii* membranes contain significant amounts of apolar lipids (Kellermann et al. 2016), this technical limitation affects the accuracy of absolute lipid concentration measurements. Analysis of isothermal compressibility (Supplementary Fig. S2B, C) revealed similar values for both lipid monolayers at surface pressures between 5–20 mN/m. Above 20 mN/m, H119 extracts exhibited slightly higher compressibility, differing by approximately 10 mN/m from WR806 extracts. Together, the monotonic nature of the isotherm curves at both 37°C and 42°C, combined with overlapping compressibility modulus values, demonstrates that both H119 and WR806 strains maintain similar membrane mechanical properties. This apparent biophysical equivalence suggests that membrane properties may not substantially contribute to differential cell biology between the strains. Notably, these findings align with a recent study on carotenoid-deficient mutants of the Gram-negative *Methylobacterium extorquens*, which similarly exhibited no significant changes in growth or membrane biophysical phenotype (Rizk et al. 2021).

We next examined living cells cultured at 42°C to assess growth dynamics and morphological characteristics, to verify and extend the results from our first publication establishing WR806 (Turkowyd et al. 2020). Growth analysis revealed comparable doubling times and growth behavior between H119 and WR806 strains in HV-Cab medium (15.2 ± 1.4 h and 13.6 ± 3.7 h, respectively; Supplementary Fig. S2D) and HV-YPC medium (11.2 ± 1.1 h and 12.4 ± 3.1 h; Supplementary Fig. S2E). When transferring cultures between media types, adaptation occurred more rapidly during the transition from HV-Cab to HV-YPC (Supplementary Fig. S2F-G), with strain-specific variations in doubling time remaining within one standard deviation.

Morphological analysis revealed that while cell circularity and aspect ratio (AR) remained consistent between strains, the cell area exhibited significant differences in both media conditions (Fig. 1D–F). WR806 cells were consistently larger, with median areas of 3.9 ± 1.5 µm² (HV-Cab) and 4.1 ± 1.5 µm² (HV-YPC), compared to H119 cells with 3.6 ± 1.2 µm² (HV-Cab) and 3.9 ± 1.4 µm² (HV-YPC) (*P* < 0.0001 for HV-Cab; *P* = 0.0002 for HV-YPC). At this point we can only speculate and may attribute this size difference to the absence of carotenoids: while our *in vitro* experiments showed membrane compressibility remains unaffected, the lack of carotenoids might influence cell volume expansion. Previous studies have found that lipidome minimalization resulted in increased cell volumes in *Mycoplasma mycoides* minimal cells, highlighting the possible effects of depleted membrane components on cell area (Justice et al. 2024). Importantly, the preserved cellular length/width ratios and circularity metrics indicate intact cell shape regulation, which is crucial for pleomorphic *H. volcanii* (Duggin et al. 2015, Patro et al. 2023). Importantly, one limitation of the direct comparison of H119 and WR806 is that the respective genetic backgrounds differ not only in the *crtI* deletion under investigation but also in the auxotrophic selection marker *hdrB*, necessary to synthesize thymidine. Previous studies showed that *hdrB* is a critical selection marker with regard to cell shape (Patro et al. 2023). However, the observed differences in cell area coincided with further differences, e.g. in shape transition and other cell shape parameters like aspect ratio and circularity, an effect that we were not able to observe in our study. Furthermore, to align the metabolic properties of H119 and WR806 as closely as possible, we conducted all comparative experiments in medium supplemented with thymidine. We assumed that H119 cells, although capable of expressing the enzymes required for thymidine synthesis, preferentially use the external supply to avoid unnecessary metabolic burden, consistent with the typical product feedback regulation observed for thymidine supply and the linked pyrimidine metabolism in other organisms, for example, in *E. coli* (Johansson et al. 2007, Lee et al. 2009). We therefore consider the strain comparison to primarily reflect the effects of *crtI* deletion on cell morphology.

In summary, these results validate WR806 as an imaging strain that maintains an almost native-like biophysical and physiological properties while enabling single-molecule detection.

### Increased control over protein expression using a novel plasmid system

Derivatives of pTA231 are often used in *H. volcanii*, which feature the constitutive promoter p.syn for POI expression and the *trpA* selection marker under p.fdx control (Allers et al. 2004, Berkemer et al. 2020). Another plasmid type used are derivatives of the plasmid pTA962, incorporating the tryptophan-inducible promoter p.tna and the selection markers *hdrB* and *pyrE2* (Allers et al. 2010). Inducible promoter systems offer significant advantages over constitutive ones, primarily by allowing precise control over expression levels. This control is particularly crucial for cell-toxic proteins, highly regulated proteins, or highly stable proteins. However, using the pTA962 system in the WR806 strain presents a limitation: Because the *trpA* deletion in WR806 disrupts internal tryptophan synthesis, cells must be continuously supplied with external tryptophan (0.25 mM) for normal growth. This couples selection and induction, as the same molecule serves both functions, resulting in constant induction of protein expression under standard growth conditions. This coupling effectively negates the benefits of an inducible system. To address this limitation, we developed a novel plasmid: pUE001 encodes the *trpA* gene, enabling internal tryptophan synthesis. Combined with p.tna for inducible POI expression, this decouples selection from induction, restoring the functionality of the inducible system while maintaining the advantages of controlled protein expression in *H. volcanii*. pUE001 was constructed by cloning the p.tna promoter from pTA962 into the backbone of pTA231, resulting in a plasmid with the *trpA* selection marker under the constitutive promoter p.fdx and a tryptophan-inducible p.tna promoter for POI expression. The plasmid was further engineered to enable labeling of POIs with a SMLM-compatible FP by inserting the coding sequence for Dendra2Hfx (Turkowyd et al. 2020) downstream of the promoter (Supplementary Fig. S1, Addgene # 234669).

To characterize the novel pUE001 expression system in biological applications we also constructed versions that encode FtsZ1:Dendra2Hfx and Cas1:Dendra2Hfx fusion proteins. These proteins were selected based on our previous challenges (data unpublished) encountered with the pTA962 expression system: The highly stable protein FtsZ1 was chosen due to its tendency to form protein aggregates and inclusion bodies which make it an ideal candidate to test for improved expression control. Cas1 was selected as a naturally low-abundant and seemingly highly regulated protein, as it had proven difficult to express in previous pTA962 fusion constructs. Both fusion proteins were checked for potential proteolysis using in-gel-fluorescence SDS-PAGE (Supplementary Fig. S4).

First, we conducted quantitative fluorescence and OD_600_ measurements of WR806 strains carrying either pUE001-Dendra2Hfx or pUE001-FtsZ1:Dendra2Hfx, using plasmid-free WR806 as a negative control. Cas1 as a lowly expressed protein (Brendel et al. 2014, Jevtić et al. 2019) was too low-abundant to be reliably measured in plate reader experiments. To characterize induction levels and fluorescence response over time, we grew each strain in HV-Cab medium with varying tryptophan concentrations (0–5 mM for WR806 carrying pUE001; 0.25–5 mM for WR806 and WR806 carrying pTA962) for 70 h. We normalized fluorescence values to optical density measurements (Fig. 2; raw data in Supplementary Fig. S3).

Generally, normalized fluorescence values of FP constructs in ensemble measurements depend on four major factors: promoter activity for protein production, FP maturation time (assumed to be constant in our conditions), protein degradation and dilution of the signal by cell division during growth (Leveau and Lindow 2001). The normalized fluorescence data (Fig. 2A, B, D, and E) reveals distinct patterns corresponding to different growth phases. During lag phase, the protein production rate (r_FP_) exceeds the cell division rate (r_Di_) and protein degradation rate (r_De_), resulting in rapidly increasing fluorescence per cell. In exponential phase, r_FP_, r_Di_ and r_De_ reach equilibrium, manifesting as a plateau in the normalized fluorescence data. When entering stationary phase, r_FP_ again exceeds r_Di_ and r_De_, leading to an increase of the fluorescence per cell.

Analysis of steady-state fluorescence intensities during exponential growth phase shows optimal induction at 3-4 mM tryptophan concentration (Fig. 2C and F), consistent with previous findings (Large et al. 2007). While pTA962-carrying strains exhibit higher absolute fluorescence intensities, pUE001-carrying strains demonstrate a more linear correlation between tryptophan concentration and expression levels. Maximum induction occurs at 3 mM for pTA962-carrying strains compared to 4 mM for pUE001-carrying strains. Crucially, since WR806 carrying pUE001 can grow without tryptophan supplementation, uninduced cultures show baseline fluorescence comparable to the empty WR806 control in exponential phase growth. This comparison of pTA962 and pUE001 steady-states during exponential growth reveals pTA962’s limited induction range when used in a strain without a chromosomal copy of *trpA*, such as WR806, at both high and low tryptophan concentrations, highlighting pUE001’s superior expression control and induction linearity. Furthermore, it indicates that pUE001 enables to make use of the low induction range between 0 and 0.25 mM tryptophan—a range that, for quite some POIs, might be crucial when aiming to study them close to their native expression levels and which we later in this work exploit for Cas1 single-molecule experiments.

Moreover, these experiments also underscore the importance of preculture conditions. All strains for these experiments measuring ensemble fluorescence were grown for about three days into stationary phase before sub-culturing, resulting in initial fluorescence intensities which are roughly equivalent to the 70-h timepoint (marked by asterixis in Fig. 2A, B, D, and E). Since WR806 carrying pTA962 requires externally supplied tryptophan for growth, it shows higher initial fluorescence than WR806 carrying pUE001 strains (asterixis in Fig. 2A, B, D, and E). This poses a major limitation for ensemble studies by precluding true zero-expression conditions. Under 0 mM tryptophan conditions, the pUE001 carrying strain showed increased fluorescence during stationary phase (Fig. 2A and D), likely due to tryptophan accumulation in the medium from sources such as degraded cell debris. This indicates that although zero-expression conditions can be achieved, the specific conditions during pre-culturing remain important.

Finally, our plate reader dataset highlights the importance of protein stability in interpreting expression level measurements. FtsZ is a filament-forming protein (Liao et al. 2021, Pende et al. 2021) whose polymerized form is largely protected from proteolytic turnover. Accordingly, studies in *E. coli* have reported an *in vivo* half-life of approximately 2 h, despite a culture doubling time of 30 min. This relationship indicates that only 13%–20% of the cellular FtsZ pool is degraded during each generation (Camberg et al. 2009, Männik et al. 2018). We thus assume that this protection effect is reflected in the higher initial values for FtsZ1: Dendra2Hfx-expressing strains of our experiments when restarting growth from preculturing stationary phase conditions (Fig. 2D and E). In contrast, strains carrying pUE001-Dendra2Hfx show a rapid decrease in fluorescence between the stationary phase and experiment start, which time-wise corresponds to a ∼1-h period between transfer to fresh medium and the start of plate-reader measurements (Fig. 2A). This effect is also absent in by 0.25 mM tryptophan continuously induced WR806 carrying pTA962 strains (Fig. 2B and E) and will be readdressed further below.

### Decoupling selection and induction enables tight expression control and reveals potential promotor-independent regulation of *trpA*

Following ensemble studies, we performed single-cell sensitive, diffraction-limited imaging of WR806 expressing three POIs: Cas1:Dendra2hfx, FtsZ1:Dendra2Hfx, and cytosolic Dendra2hfx, using both pUE001 and pTA962 expression systems. This approach enabled detection of the low-abundant Cas1 protein (Brendel et al. 2014, Jevtić et al. 2019). Notably, we found that plasmid-based expression of fusion proteins is not universally successful, as demonstrated by our attempts to express FtsZ1:Dendra2Hfx from pTA231 under the constitutive p.syn promoter (Supplementary Fig. S5), although cytosolic fluorescent proteins express successfully (Turkowyd et al. 2020). Similar expression challenges have been reported elsewhere (Ithurbide et al. 2024). We have not investigated this further but recommend to check combinations of imaging strains and expression systems carefully for each POI and experimental set up.

**Figure 3:**
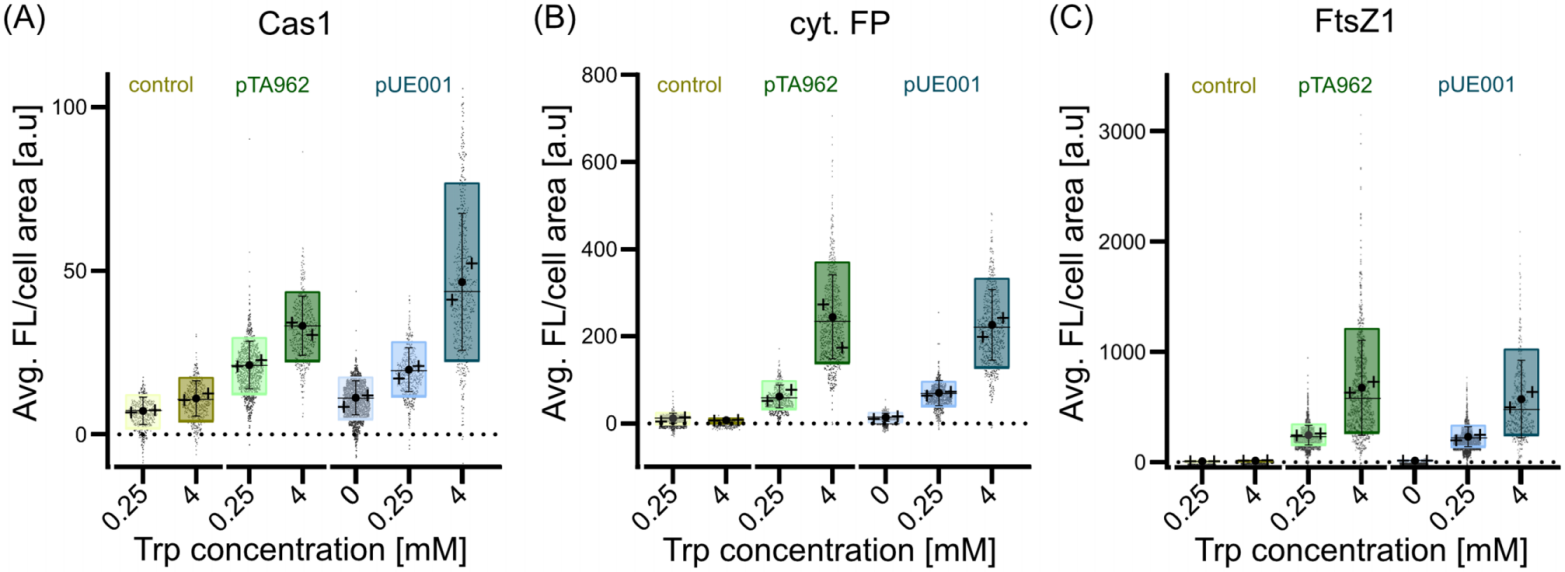
Single-cell analysis of Dendra2Hfx fluorescence expression in H. volcanii strain WR806. Quantification of background-corrected green fluorescence intensity per cell area using diffraction-limited fluorescence microscopy. Cells expressed different Dendra2Hfx constructs from either pTA962 (green, two middle data sets in each panel) or pUE001 (blue, three right data sets in each panel) plasmids in three configurations: (A) Cas1: Dendra2Hfx fusion protein, (B) cytosolic Dendra2Hfx, and (C) FtsZ1: Dendra2Hfx fusion protein. Cells were inoculated at an OD_600_ of 0.1 and cultured under inducing conditions for 18 h, allowing them to re-enter exponential growth phase and reach steady-state equilibrium between fluorescence production and growth rate, as previously demonstrated in Fig. 2. Plasmid-free WR806 (yellow, two left data sets in each panel) served as negative control. Data from biological duplicates (n > 1000 cells total) are shown as individual data points and summarized in box plots showing overall mean (circle), individual replicate means (crosses), median (horizontal line), 10_th_–90_th_ percentile range (box), and standard deviation (whiskers).

We cultured strains with high (4 mM) and low (0.25 mM) tryptophan concentrations, with WR806 pUE001 strains additionally grown without tryptophan supplementation. Imaging after 18 h in exponential phase demonstrated successful expression of all POIs using pUE001 and confirmed reliable p.tna promoter induction, independent of the plasmid-encoded selection marker *trpA* (Fig. 3). This data reinforces our plate reader findings and extends them to Cas1:Dendra2Hfx expression. While pTA962 cannot be grown without constant p.tna promoter induction as seen by leaky expression across all POIs, pUE001’s decoupled induction resulted in tight expression control under tryptophan-free conditions (Fig. 3). This feature allows for fine-tuned expression at tryptophan concentrations between 0–0.25 mM of highly regulated, toxic, or growth-inhibiting proteins, including CRISPR-Cas components (Fig. 3A) and stabilized proteins like FtsZ1 (Fig. 3C). Intriguingly, since *trpA* on pUE001 is under control of the constitutive p.fdx promoter, our results support our hypothesis of promoter-independent regulation (e.g. known for other organisms, e.g. *E. coli* using attenuation and product feedback control), though we did not investigate this mechanism further.

In addition, this data reveals protein-specific expression patterns, particularly evident in comparing average intensity levels between Cas1:Dendra2Hfx and FtsZ1:Dendra2Hfx expressing strains (Fig. 3A and C). Furthermore, we can assume that cytosolic Dendra2Hfx appears to minimally interfere with cellular processes, making it a potential benchmark to estimate further unregulated protein abundance levels of pUE001 expression in *H. volcanii*.

Notably, high tryptophan concentrations (4–5 mM) induced some autofluorescence in WR806 (Fig. 3A), with average intensities increasing from 7 ± 4 a.u. in WR806 0.25 mM to 11 ± 5 a.u. in WR806 4 mM above background. This autofluorescence increase upon high tryptophan concentrations might pose a problem under SMLM conditions. However, high tryptophan concentrations of 4 mM are typically not used for imaging cell biology as severe POI overproduction would most likely show alterations. Furthermore, OD measurements of WR806 under high tryptophan concentrations show accelerated entry into stationary phase (Supplementary Fig. S3C, H). This effect might be related to indole, the primary product of tryptophan degradation by *tnaA*-encoded tryptophanase as known for Gram-negative bacteria and halophilic archaea (Boya et al. 2021). Indole has been shown to function as a signaling molecule affecting biofilm formation, cell division, and virulence in prokaryotes (Di Martino et al. 2003, Lee and Lee 2010), including persister cell formation in *H. volcanii* (Megaw and Gilmore 2017). Studies in *E. coli* have shown indole’s interaction with the stationary phase-inducing sigma factor δ^S^ (Wang et al. 2001, Lee and Lee 2010, Joffré et al. 2022) and transient spikes in intracellular indole concentration during exponential-to-stationary phase transition (Gaimster et al. 2014). Similar mechanisms might exist in *H. volcanii*. While the global effect of indole accumulation on cellular processes may explain cellular stress and thus, next to increased fluorescence background by aromatic tryptophan itself, might contribute to autofluorescence at high tryptophan concentrations, the overall increase in autofluorescence in our hands remains negligible when imaging of FP-expressing cells. Comparing the autofluorescence levels of WR806 with signal of low-abundant proteins like Cas1 at 4 mM tryptophan concentrations, the FP signal still shows an about four-times increased signal with a mean fluorescence intensity of 47 ± 21 a.u.. For FtsZ1 this difference is even more pronounced, as the signal is about 50 times higher with a mean fluorescence intensity of 571 ± 351 a.u. (Fig. 3).

### Tryptophan synthesis by *trpA* can prevent inclusion body formation in WR806 carrying pUE001 expression constructs

**Figure 4:**
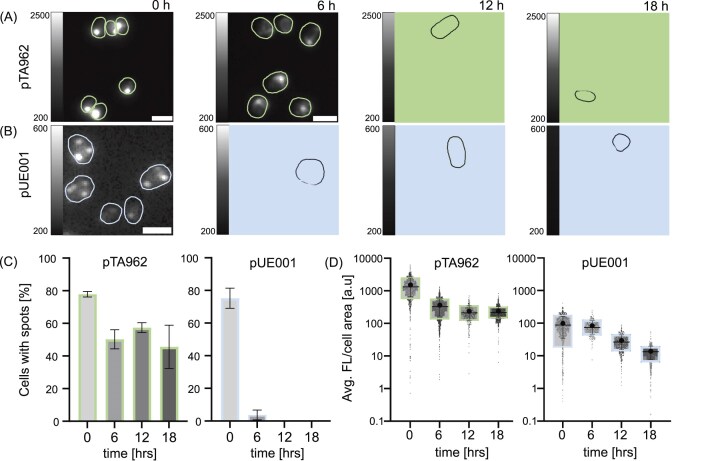
Time-course analysis of FtsZ1:Dendra2Hfx localization and expression in *H. volcanii* strain WR806. Cells were taken from a three-day stationary pre-culture supplied with 0.25 mM tryptophan in the case of WR806 pTA962 and 0 mM tryptophan in the case of WR806 pUE001, inoculated under the same conditions and imaged after 0, 6, 12, and 18 h by diffraction-limited epifluorescence microscopy (18 h samples are the same dataset as in Fig. 3). Measurements were taken in biological duplicates. (A, B) Representative fluorescence images showing green Dendra2Hfx fluorescence signal in quantitative grayscale intensities. Scale bar 2 µm. (C) Mean fluorescence intensity per cell area plotted on logarithmic scale. (D) Percentage of cells displaying fluorescent spots (in addition to FtsZ1 ring structures).

Conducting the single-cell diffraction-limited imaging as shown in Fig. 3, indicated that handling of precultures and growing conditions critically affects imaging outcomes. We frequently observed fluorescent foci and higher cellular fluorescence intensities WR806 strains expressing FtsZ1:Dendra2Hfx (Fig. 4A and B), even though the cultures were regrown for 18 h after sub-culturing from stationary phase precultures. To further investigate the accumulation of fluorescence during stationary phase (Fig. 2) and formation of fluorescent foci (Fig. 4A and B), we quantified the number of fluorescent spots per cell over time (Fig. 4C). We used minimal tryptophan concentrations for each strain: 0 mM for pUE001-FtsZ1:Dendra2Hfx and 0.25 mM for pTA962-FtsZ1:Dendra2Hfx and re-inoculated the strains from three-days stationary phase pre-cultures. Fluorescent spots were counted at 0, 6, 12, and 18 h. Cultures measured directly from stationary phase precultures at 0 h showed similar proportions of cells with fluorescent spots: 75 ± 6% for pTA962 and 77.8 ± 2% for pUE001 strains (Fig. 4C). Upon sub-culturing into fresh medium, we observed distinct temporal patterns: WR806 carrying pUE001 showed complete elimination of fluorescent foci after 12 h, while WR806 carrying pTA962 maintained a minimum of 45 ± 13% cells with spots even after 18 h (Fig. 4C), the condition which was measured in Fig. 3. This decrease of cells exhibiting spots correlated with the declining average cellular fluorescence intensities (Fig. 4D). Notably, spot size and intensity differed drastically between strains: WR806 carrying pTA962 typically showed single, larger, and brighter foci per cell, while WR806 carrying pUE001 exhibited multiple smaller spots that were an order of magnitude less intense (Fig. 4A and B).

Given their brightness and persistence we thus speculate that foci in WR806 carrying pTA962 represent inclusion bodies (IBs) and result from constitutive protein expression under normal growth conditions. IBs, typically associated with protein overproduction and cellular stress (Fahnert et al. 2004, Landgraf et al. 2012, Kopp et al. 2023), pose significant challenges for both biotechnology applications and fluorescence imaging by deviating from native cell biology and compromising the applications. In contrast, the transient spots in the pUE001 carrying strain likely might represent smaller FtsZ1 oligomers. During stationary phase, when cell division is arrested, overexpressed FtsZ1 might form these short filaments, which subsequently could be incorporated into functional FtsZ1 rings upon resumption of cell growth and cell division. This process is supported by the images indicating a FtsZ1:Dendra2Hfx redistribution from small spots to distinct mid-cellular rings between 0 and 6 h after restarting growth, where average fluorescence intensity is maintained but reorganized protein localization shows a clear shift (Fig. 4B). As cultures enter exponential phase, per-cell fluorescence intensity decreases, consistent with our plate reader data (Fig. 2). The remarkable stability of FtsZ1 is further demonstrated by the detection of faint FtsZ rings even after 18 h without induction in WR806 pUE001-FtsZ1:Dendra2Hfx, likely due to stochastic p.tna promoter activity (Fig. 4B).

We also observed fluorescent spots, albeit at much lower frequencies, in strains expressing Cas1:Dendra2Hfx (0.75% of cells for pTA962 and 0.15% for pUE001, data not shown), while strains expressing cytosolic Dendra2Hfx never showed foci. This protein-specific aggregation pattern suggests a strong correlation between protein function and aggregation propensity, while confirming Dendra2Hfx as an ideal FP for *H. volcanii* studies due to its minimal interference with cellular processes.

These findings emphasize the importance of careful strain, expression system, and induction parameter selection based on the POI. Furthermore, they demonstrate the superior performance of the pUE001 system in terms of rapid adaptation to non-inducing conditions and overall experimental utility.

### Cas1 might be involved in UV-light-associated repair of DNA damage

Building on our ensemble measurement results, we next turned to the more technically challenging regime of single-molecule imaging, focusing on the low-abundant protein Cas1. Establishing optimal conditions for imaging Cas1 dynamics allowed us to both demonstrate the high performance of our imaging system and directly address a current cell biological question in the field about the cellular functions of the native CRISPR-Cas type I-B system of *H. volcanii*. CRISPR-Cas systems, initially hypothesized to function in DNA repair before their role in adaptive immunity was discovered (Makarova et al. 2002), are increasingly recognized for their diverse cellular functions. Recent studies have revealed strong interconnections between Cas proteins and fundamental cellular processes, including gene regulation, virulence, signal transduction, and DNA repair mechanisms (Faure et al. 2019). The adaptation complex protein Cas1, with its nuclease activity, demonstrates particular utility in DNA repair through its ability to cleave branched DNA substrates. This activity has been documented across domains, from *E. coli* to archaeal *H. volcanii*, suggesting a conserved link between DNA repair and Cas1 function (Babu et al. 2011, Rollie et al. 2015, Wörtz et al. 2022), summarized in Supplementary Table S6. Importantly, previous studies showed Cas1 and the flap endonuclease Fen1 have redundant roles in repairing UV-light-induced DNA damage, as double-deletion mutants show significantly reduced survival rates (Wörtz et al. 2022). Furthermore, the reported direct interaction between Cas1 and nucleotide excision repair (NER) proteins associated with UV damage repair in *H. volcanii* (Wörtz et al. 2022), along with an increased UV sensitivity in *E. coli* Cas1 deletion strains and *H. volcanii* Cas1 and Fen1 deletion strains (Babu et al. 2011, Wörtz et al. 2022), prompted us to investigate Cas1’s role in UV-induced DNA repair using sptPALM. Our primary aim in this first biological application of pUE001 was to provide first visualization of Cas1 molecules being involved in the UV-induced DNA damage response. As discussed in the next paragraph, we thus leveraged the robust pUE001 expression for low-abundant proteins, and quantified the dynamic molecular Cas1 response on physiologically meaningful UV damage levels and time-scales, adding imaging evidence to previous biochemical and genetic literature data of Cas1 involvement into DNA repair.

Our novel pUE001 plasmid, which demonstrates superior performance compared to pTA962 in WR806, enables precise control of expression levels between 0 and 0.25 mM tryptophan—a crucial advancement for sensitive sptPALM measurements. To investigate molecular Cas1 dynamics after UV-induced DNA damage using sptPALM, we chose to express pUE001-Cas1:Dendra2Hfx in WR806. Importantly, the strain also encodes for the native, untagged genomic Cas1, as studies indicate that expressing tagged POIs in their respective deletion strains can significantly disrupt cellular function (Duggin et al. 2015, Liao et al. 2021). Cultures were kept in early exponential phase preparatory to imaging to ensure cells were in a healthy growth condition, where r_FP_, r_Di_ and r_De_ are in equilibrium and heterogeneity in induction levels, e.g. due to cell debris can be neglected (Fig. 2). Given the low abundance and tight regulation of Cas proteins (Brendel et al. 2014), this approach, combined with minimal induction (0.25 mM tryptophan), ensured the required low POI expression and thus native-like functional behavior of defense and possible DNA repair mechanisms.

**Figure 5:**
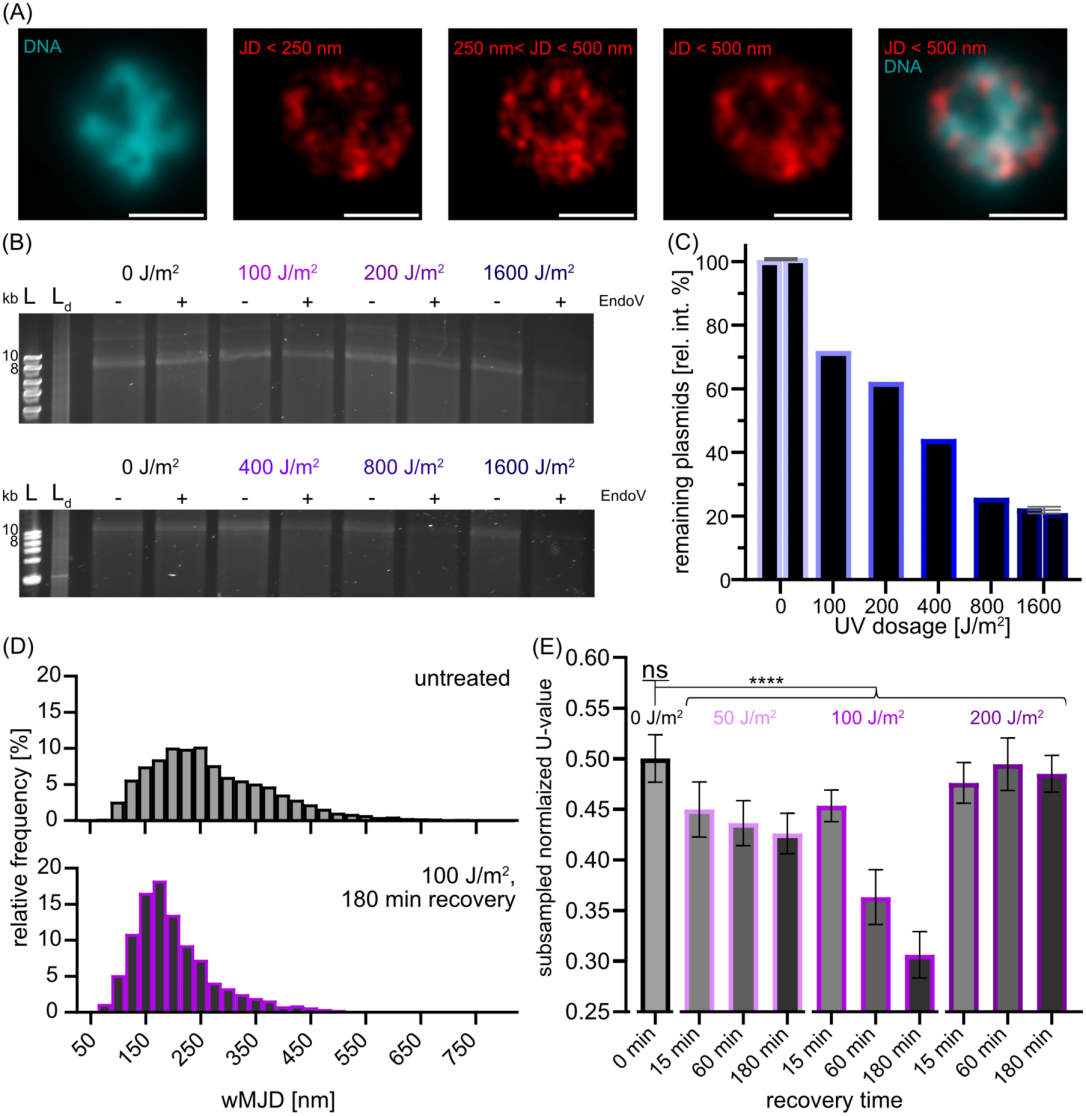
Analysis of Cas1:Dendra2Hfx subcellular localization and single-molecule dynamics in response to UV-light damage in *H. volcanii* strain WR806 pUE001. (A) Exemplary image of an untreated WR806 pUE001-Cas1:Dendra2Hfx expressing cell that was stained with Hoechst 33342 to visualize DNA. Cas1:Dendra2Hfx dynamics were measured using sptPALM microscopy. Trajectories of Cas1:Dendra2Hfx were tracked and Cas1 localizations were separated between the apparent dynamic populations of trajectories with slow dynamics (JD < 250 nm) and faster dynamics (JDs of 250–500 nm). The last image shows a composite image of all subcellular Cas1 positions from both diffusive populations (trajectories with JD < 500 nm). All Cas1 sub-cellular positions were visualized according to their localization precision using a Gaussian blur filter (Endesfelder et al. 2014), DNA images were deconvolved. Scalebar 1 µm. (B) Denaturing urea agarose gel electrophoresis of pUE001-Dendra2Hfx plasmid DNA isolated from WR806 cells immediately after UV-damage treatment. Cells were treated with doses of 0 J/m^2^, 100 J/m^2^, 200 J/m^2^, 400 J/m^2^, 800 J/m^2^, and 1600 J/m^2^ of UV irradiation and DNA was treated with T4 Endonuclease V to digest CPD lesions. To normalize the Endo V treated conditions (+), respective equal amounts of untreated plasmid DNA (-) were also applied to the gel. Ladder: NEB 1 kb ladder; L_d_: denatured ladder. (C) Quantification of remaining plasmid DNA after UV treatment with increasing doses using the band intensities from (B). The normalized intensities of the treated bands (+) were calculated based on the respective untreated samples (−). 0 J/m^2^ and 1600 J/m^2^ samples were applied to both agarose gels as a quantitation error control, and mean and SD were calculated (horizontal lines and whiskers). (D, E) sptPALM data of Cas1:Dendra2Hfx in WR806. Exponentially growing cells were induced with 0.25 mM tryptophan for inducing Cas1 protein expression and exposed to UV-light radiation at 265 nm at 0, 50, 100, or 200 J/m² and measured after a recovery time of 0, 15, 60, and 180 m post-exposure. (D) Distribution of Cas1 dynamics comparing untreated cells with cells showing maximal response (180 m recovery after 100 J/m² UV exposure). Dynamics are quantified by weighted mean jump distances (wMJD) from individual single-molecule trajectories across two biological replicates. (E) Statistical comparison of all experimental conditions to the untreated control using subsampling of each data set and comparing subsampled data using the Mann–Whitney-U-test normalized to the respective sample size. The untreated data was split into two halves and tested against each other as a control. Bars represent the mean normalized U-value determined by subsampling, error bars represent the 5% and 95% confidence intervals. All wMJD distributions are provided in Supplementary Fig. S8, normalized U-value distributions determined by subsampling are shown in Supplementary Fig. S9. ns indicates not significant, **** indicates *P* < 0.0001.

Using dual-color imaging, we obtained a first visual impression of DNA-Cas1 interactions in untreated cells by examining Cas1 localization relative to DNA-rich regions (Fig. 5A and Supplementary Fig. S6). To achieve high-resolution images of intracellular DNA, 3D stacks were acquired and deconvolved using the setup-specific point spread function of our microscope to computationally mitigate the effects of light diffraction on image resolution. Overlaid composite images of deconvolved DNA and Cas1:Dendra2Hfx signals show that Cas1 predominantly localizes to the periphery of DNA-dense regions, whereas regions with low DNA occupancy show little to no detectable Cas1 signal. These defined localization patterns visualize Cas1’s general DNA-interacting activity and suggest that the majority, if not all, of Cas1 molecules frequently interact with DNA, independent of any DNA damage. This observation is in line with the protein’s role in the adaptation complex of *H. volcanii* (Stachler et al. 2017, 2020).

Next, we revisited our aim of investigating the role of Cas1 in NER beyond the data from previous publications. To this end, we induced cyclobutane pyrimidine dimers (CPDs)—the predominant form of UV-associated DNA damage—at thymidine dimer target sites in live WR806 pUE001-Cas1:Dendra2Hfx cells using 265 nm UV light. Similar treatments have been reported to induce DNA lesions (McCready 1996, Stantial et al. 2016), summarized in Supplementary Table S7. To quantify the efficiency of CPD-formation, we isolated and linearized plasmid DNA from treated cells and then digested the DNA with T4 Endonuclease V (Fig. 5B, C). As T4 Endonuclease V specifically cuts CPDs, the reduction in intensity of the plasmid DNA band on agarose gels directly reflects the fraction of plasmids that remain intact, providing a quantitative measure of DNA lesion accumulation upon increasing UV-light dosages. We applied UV-light dosages of 0, 100, 200, 400, 800, and 1600 J/m^2^ and observed an exponential decline of remaining, intact plasmid DNA (data fitted with an exponential decay, Supplementary Fig. S7, decay constant K = 3×10^−3^ m^2^/J). This is consistent with a Poisson process in which absorbed photons independently induce strand breakage. Doubling the UV dosage doubles the expected number of breaks, producing the characteristic exponential decay of the intact DNA band, which corresponds to plasmids that have so far experienced no strand breakage from the inducing UV light—the Poisson “zero-event” case.

It should be noted that this quantification represents a conservative estimate, as the pUE001-Cas1:Dendra2Hfx plasmid contains, on average, one potential thymidine dimer target site per 9.1 nucleotides. Fragments with lesions occurring near the ends of the linearized plasmid strand cannot be distinguished from the intact DNA. To further validate our UV-damage assay, we monitored growth of WR806 pUE001-Cas1:Dendra2Hfx strains after UV treatment (HV-CAB, non-expressing conditions with 0 mM tryptophan) and observed a dose-dependent reduction in exponential-phase growth rates and stationary-phase OD values, consistent with the applied UV dosages (Supplementary Fig. S10) and confirming that the selected doses fall within a physiological range in which cells remain viable while initiating DNA repair.

Next, we introduced UV-induced DNA lesions into WR806 pUE001-Cas1:Dendra2Hfx cells using physiologically relevant low (50 J/m²), medium (100 J/m²), and high (200 J/m²) doses of 265 nm UV light, based on previous studies of the UV damage-associated NER uvrABCD system in *H. volcanii* (Lestini et al. 2010), and followed the dynamics of Cas1:Dendra2Hfx at 15, 60, and 180 m post-exposure. As measured in *in vivo* single-molecule studies of CRISPR-Cas interference, both, Type-II Cas9 (Knight et al. 2015, Jones et al. 2017, Martens et al. 2019) and Type-I Cascade complexes (Turkowyd et al. 2019, Vink et al. 2020) exhibit rapid diffusion and transient DNA binding while scanning the DNA for protospacer adjacent motif (PAM) sites. Similar dynamics can be assumed for Cas1 in *H. volcanii*, as the protein takes over a major function in PAM verification during adaptation (Nuñez et al. 2014, Wang J et al. 2015). Once Cas1 becomes actively involved in e.g. DNA defense or DNA repair processes, dwell times on target DNA sites are expected to increase, resulting in a measurable slowdown of its otherwise fast-diffusive dynamics.

Analysis of weighted mean jump distances (wMJD) revealed significant UV damage-induced slowdown of a mobile fraction of Cas1 (*P* < 0.0001) across all conditions (Fig. 5E and Supplementary Fig. S8). Normalized U-values demonstrate both, time- and dose-dependent responses, with lower U-values indicating stronger effects. In low UV exposure conditions, we observed a gradual mobility decrease, with mean U-values declining from 0.45 (15 min) to 0.43 (180 min). The decline in mean U-values was increased for medium UV exposure conditions (100 J/m²) with values decreasing from 0.45 (15 min) to 0.31 (180 min), indicating a more severe damage response in line with the increased number of damaged DNA sites. Generally, the most pronounced response occurred after a medium exposure and 180 min (Fig. 5D and E). Interestingly, high UV doses (200 J/m²) produced an initial slowdown at 15 min followed by a shift back to match more closely again to the original distribution at 60 and 180 min. Cytosolic Dendra2Hfx as a control did not show any change in diffusive behavior (data not shown).

We also explored the subcellular localization patterns of fast and slow diffusing Cas1:Dendra2Hfx (Fig. 5A). Based on the wMJD histograms, we split all trajectory data into the slowly diffusive population of Cas1 with jump distances (JD) of under 250 nm, and fast diffusing Cas1 with JDs from 250 and 500 nm. Visualization of both populations revealed that both populations represent Cas1 colocalizing with DNA, with a majority of Cas1 molecules residing at the periphery of DNA-dense areas. This holds true for both diffusive states of Cas1 which underlines our hypothesis Cas1 molecules consistently interacting with DNA.

As our experiments do not directly visualize interactions between Cas1 and DNA repair proteins, we can only hypothesize about the underlying mechanisms explaining the observed dynamics. The timing of Cas1 slowdown differs from *E. coli*’s rapid NER response, where uvrAB proteins react within 5–15 min of UV damage (Stracy et al. 2016). However, with regard to the comparably slow generation turnover of *H. volcanii*, it seems plausible that a UV damage response might occur later if e.g. coupled to transcription processes (Savery 2007, Pérez-Arnaiz et al. 2020). Interpretation of Cas1 dynamics at 200 J/m² requires caution, as this dose exceeds previously reported UV exposures for *H. volcanii* [maximum 100 J/m² (Lestini et al. 2010, Wörtz et al. 2022)] and may trigger temporary stagnation of cell proliferation and/or in ultimate cell death, with both conditions diminishing any metabolically-coupled Cas1 response. Additionally, other DNA damage repair systems, e.g. for double-strand-repair (Pérez-Arnaiz et al. 2020), might be differently involved and/or prioritized for different, i.e. higher UV dosages. Double-strand breakages are a more severe form of DNA damage when compared to CPDs and are known to result from strong UVC light (Rolfsmeier et al. 2010). Thus, NER-associated slowdown of Cas1 might occur later and/or less prominent. While technical constraints prevented observations earlier than 15 min post-UV-exposure, the gradual response patterns at lower doses suggest maximum Cas1 slowdown may occur beyond 180 min in these conditions.

### Conclusions

The study of molecular cell biology through fluorescence imaging techniques as a direct, visual approach depends on labeling systems that minimally perturb native cellular processes. This requirement becomes particularly detectable in single-molecule imaging, where even subtle alterations can significantly impact observations. Given the still limited availability of established expression systems and selection markers in the archaeal model organism *H. volcanii*, we developed and characterized a novel expression system optimized for the autofluorescence-free imaging strain WR806.

Our work validates that the combination of the carotenoid-free strain WR806 and the novel pUE001 expression plasmid is an excellent tool for quantitative and low-induction fluorescence studies of live *H. volcanii* cells as the biophysical and physiological parameters remain unaffected by carotenoid depletion upon deletion of *crtI* and protein expression from pUE001 prevents artefacts such as inclusion body formation, and enables controlled and finetuned expression. We demonstrate through both ensemble and single-cell fluorescence measurements that decoupling induction from selection is vital to obtain robust data which closely reflects native cell biology. Our system enables precise control of protein expression levels for diverse proteins of interest—Cas1:Dendra2Hfx, FtsZ1:Dendra2Hfx, and cytosolic Dendra2Hfx—with fine-tunable regulation through tryptophan concentrations. This tight control represents a significant advancement over the previously used pTA962 system. Furthermore, our system offers practical utility, as expression levels can be effectively reset through the sub-culturing of precultures.

Our findings establish that providing a copy of *trpA* is essential for proper control of the tryptophan-inducible p.tna promoter. More broadly, our work emphasizes the critical importance of experimental design and implementation when combining selection markers, promoter systems, and strains, particularly in *H. volcanii*, which exhibits complex and sensitive pleomorphic characteristics (Patro et al. 2023). This strategic approach to expression system design proves especially crucial for maintaining near-native cellular conditions in molecular imaging studies.

We then investigated the interaction between native CRISPR-Cas Type I-B systems and DNA repair in *H. volcanii*. We observed a significant, dose- and time-dependent reduction in Cas1 protein mobility following UV-light-associated DNA damage, with maximum response at 100 J/m² after 180 min recovery, suggesting interplay between UV damage repair mechanisms and CRISPR-Cas proteins. The involvement of Cas proteins in DNA repair has been documented across diverse organisms (Faure et al. 2019). Cas1 specifically demonstrates nuclease activity on branched DNA substrates, particularly 5'-flaps, as shown *in vitro* using purified proteins from *E. coli* and *Sulfolobus solfataricus* (Babu et al. 2011, Rollie et al. 2015). This activity on structures commonly associated with DNA repair intermediates suggests a role for Cas1 in DNA maintenance. In *H. volcanii*, Cas1 exhibits functional similarity to the flap exonuclease Fen1 and associates with several replication and repair proteins, including the double-strand break repair protein Rad50, DNA mismatch repair MutS, and DNA helicase MCM (Wörtz et al. 2022).

Particularly relevant to our findings, Cas1 co-purifies with nucleotide excision repair (NER) proteins UvrA, UvrB, and UvrD (Wörtz et al. 2022), which are known mediators of UV-induced DNA damage repair (Lestini et al. 2010). This association aligns with observations in *E. coli*, where *cas1* deletion increased UV sensitivity. Notably, double mutants of *cas1* and *uvrABC* homologues *ruvA, ruvB*, and *ruvC* show no additional UV sensitivity increase, suggesting the respective proteins operate in a shared pathway (Babu et al. 2011). Wörtz et al. (2022) proposed that recruitment of Cas1 to DNA repair complexes at damage sites might prevent Cas1-Cas2 adaptation complex formation, thereby inhibiting auto-immunity and positioning Cas1 as an accessory protein in DNA damage repair—a hypothesis supported by our observations.

Our findings reinforce the emerging view of DNA repair, replication, and defense as interconnected, dynamic networks, which highlights the expanded possible roles for CRISPR-Cas I-B systems beyond immunity. This work provides evidence for Cas1’s involvement in UV-light-induced DNA damage response through SPT data, complementing previous biochemical and genetic studies.

## Resources

All pUE001 plasmids are available on Addgene: #234 669 (Dendra2Hfx), #234 670 (Cas1:Dendra2Hfx), #234 671 (FtsZ1:Dendra2Hfx).

## Supplementary Material

uqag014_Supplemental_File

## Data Availability

All data underlying the figures and supplementary figures is available under ZENODO (10.5281/zenodo.14845238).
