# Supplementary material for: A novel expression system for imaging single-molecule fluorescence in Haloferax volcanii WR806 enables visualization of altered Cas1 dynamics during UV-induced DNA damage response
Source: Microlife. 2026 Apr 13;7:uqag014. doi: 10.1093/femsml/uqag014 (PMC13147458; doi:10.1093/femsml/uqag014)
Supplement: uqag014_Supplemental_File [file uqag014_supplemental_file.pdf]

Supplementary data to

A novel expression system for imaging single-molecule fluorescence in *Haloferax volcanii* WR806 enables visualization of altered Cas1 dynamics during UV-induced DNA damage response

Paula R Schrage[1], Uliana Afonina[1,2], Julia Wörtz[3], Anita Marchfelder[3], Koen JA Martens[1,§] James Sáenz[2] and Ulrike Endesfelder[1]

Supplementary Table S1 – S7

Supplementary Figure S1 – S10

Supplementary Table S1. Strains used in this work.

Strain	Genotype	Needed Additives	Reference
NEB® 5-alpha Competent *E. coli* (High Efficiency)	*fhuA2Δ(argF-lacZ)U169 phoA glnV44 Φ80Δ(lacZ)M15 gyrA96 recA1 relA1 endA1 thi-1 hsdR17*	-	New England Biolabs (Frankfurt am Main, Germany)
dam⁻/dcm⁻ Competent *E. coli*	*ara-14 leuB6 fhuA31 lacY1 tsx78 glnV44 galK2 galT22 mcrA dcm-6 hisG4 rfbD1 R(zgb210::Tn10)* Tet^S *endA1 rspL136* (Str^R) *dam13::Tn9* (Cam^R) *xylA-5 mtl-1 thi-1 mcrB1 hsdR2*	-	New England Biolabs (Frankfurt am Main, Germany)
H. volcanii WR806	DS70-wildtype (ΔpHV2), Δ*pyrE2*, Δ*trpA*, Δ*leuB*, Δ*hdrB*, Δ*crtI*	Thymidine, uracil, tryptophan	(Turkowyd et al., 2020)
H. volcanii H119	DS70-wildtype (ΔpHV2), Δ*pyrE2,* Δ*trpA*, Δ*leuB*	Tryptophan, uracil	(Allers et al., 2004)

Supplementary Table S2. Oligonucleotides used in this work.

Oligonucleotide	Sequence 5’ → 3’	Application
screening_pUE001_for	AGTGAGCGAGGAAGCGGAAG	Verification of positive transformants
screening_pUE001_rev	GTGGCGAGAAAGGAAGGGAAG	Verification of positive transformants
sequencing_Dendra_rev	TCGCCTTCGATGACGAACGC	Verification of positive transformants
pTA231_cas1_dendra_fwd	ccatcgcatttttcggcgcgAAGCTTGGTACCGATATCGAATTCGATATCAAGC	Backbone amplification pUE001-cas1:Dendra2Hfx, pUE001 ftsZ1:Dendra2Hfx
pTA231_assembly_dendra_rev	GGATCCGAGCTCGCGGCC	Backbone amplification pUE001-cas1:Dendra2Hfx, pUE001 ftsZ1:Dendra2Hfx
cas1_Dendra_fwd	gcggccgcgagctcggatccGACTTCGACGACTACTTCGAC	Insert amplification cas1, ftsZ1
cas1_Dendra_rev	CGCGCCGAAAAATGCGATG	Insert amplification cas1, ftsZ1
pUE001-Dendra2Hfx_fwd	gcggacctattgcgcatatgaacacgccgggcatcaac	Vector amplification pUE001-Dendra2Hfx
pUE001-Dendra2Hfx_rev	CATATGCGCAATAGGTCCGC	Vector amplification pUE001-Dendra2Hfx

Supplementary Table S3. Vectors used in this work.

**Vector name**	**Features**	**Reference**
pTA231-p.Syn-Dendra2Hfx	p.syn promoter, *trpA*, *ampR,* encodes cytosolically expressed Dendra2Hfx, shuttle vector	(Turkowyd et al., 2020)<br>Addgene: #164660
pTA962-Dendra2Hfx	p.tna promoter, *hdrB*, *pyrE*2, *ampR*, encodes cytosolically expressed Dendra2Hfx, shuttle vector	This work
pTA962-FtsZ1:Dendra2Hfx	p.tna promoter, *hdrB*, *pyrE*2, *ampR*, encodes FtsZ1:Dendra2Hfx fusion protein, shuttle vector	(Turkowyd et al., 2020)
pTA962-Cas1:Dendra2Hfx	p.tna promoter, *hdrB*, *pyrE*2, *ampR*, encodes Cas1:Dendra2Hfx fusion protein, shuttle vector	(Wörtz, 2022)
pUE001-Dendra2Hfx	p.tna promoter, *trpA*, *ampR,* encodes cytosolically expressed Dendra2Hfx, shuttle vector	This work<br>Addgene # 234669
pUE001-Cas1:Dendra2Hfx	p.tna promoter, *trpA*, *ampR,* encodes Cas1:Dendra2Hfx fusion protein, shuttle vector	This work<br>Addgene # 234670
pUE001-FtsZ1:Dendra2Hfx	p.tna promoter, *trpA*, *ampR,* encodes FtsZ1:Dendra2Hfx fusion protein, shuttle vector	This work<br>Addgene # 234671

Supplementary Table S4. Parameters used in ThunderSTORM 1.3 for localization of single emitters

Analysis filter	WaveletFilter (B-spline)<br>scale: 2.0<br>oder: 3
Analysis detector	Local maximum<br>connectivity: 8<br>threshold: 1.4*std.(Wave.F1)
Analysis estimator	PSF: integrated gaussian<br>fitting redius: 3<br>method: max. likelihood<br>initial sigma: 1.6<br>full image fitting: false<br>mfaEnabled: false<br>nMax: 0<br>pVAlue: 0.0<br>keepSameIntensity: false<br>intensityInRange: false
Post processing	[]
Is 3D	false
Is set 3D	true

Supplementary Table S5. Parameters used in *swift v0.4.3* to determine trajectories of single molecules.

diffraction_limit	150
directed_motion	false
exp_displacement	400
exp_noise_rate	10
max_blinking_duration	2
max_displacement	2.5 x exp_displacement
max_displacement_pp	3.5 x exp_displacement
max_log_complexity	13
max_memory	500
max_particle_count	2
max_subgraph_size	50000
p_bleach	0.1
p_blink	0.001
p_reappear	0.5
p_switch	0.001
precision	40
precision_z	100
pruning_base	1.5
pruning_rate	0.2
random_seed	42
threads	12
w_diffusion	2
w_dir_diffusion	1
w_immobile	1

Supplementary Table S6. Literature overview of data indicating Cas1 involvement in DNA repair in *H. volcanii, E. coli* and *Sulfolobus solfataricus*.

Organism	Main finding	Method	Source
H. volcanii	Cas1 complementation rescues cell survival after H_2O_2 induced DNA damage in Cas1 deletion strains	Growth assay	(Wörtz et al., 2022)
H. volcanii	Cas1 and Fen1 have a redundant role in UV light induced DNA damage repair, as indicated by a significantly reduced survival rate of double-deletion strains when compared to the respective single deletion strain	Growth assay	(Wörtz et al., 2022)
H. volcanii	Cas1 interacts with UV damage repair proteins of the nucleotide excision repair system UvrA, UvrB and UvrD	Pull-down assay with FLAG-Cas1	(Wörtz et al., 2022)
H. volcanii	Cas1 processes 5' flap DNA substrates similar to Fen1	*In vitro* processing of 5' flap DNA substrate visualized via denaturing PAGE	(Wörtz et al., 2022)
E. coli	Cas1 (YgbT) processes different (branched) DNA substrates	*In vitro* processing of different branched substrates visualized via denaturing PAGE/native PAGE	(Babu et al., 2011)
E. coli	Deletion of Cas1 (YgbT) increases sensitivity to UV treatment.resubstitution of catalytically dead Cas1 does not restore survival	Growth assay	(Babu et al., 2011)
S. solfataricus	Cas1 processes flapped and branched DNA substrates	Denaturing PAGE	(Rollie et al., 2015)

Supplementary Table S7. Literature overview of UV-light induced DNA damage assays in *H. volcanii.*

Main finding	Method	Source
CPD damage and repair of the lesions in transcribed and non-transcribed strands of a genomic operon after UV damage in H26 red lab strain	256 nm UV lamp on a shallow layer of cells in a petri dish, 65 J/m^2; EndoV digest	(Stantial et al., 2016)
CPD and 6-4-photoproducts damage and repair induced by UV-light	Undefined "short" wavelength, 50 J/m^2; 6-4-photoproduct and CPD specific immune assay	(McCready, 1996)

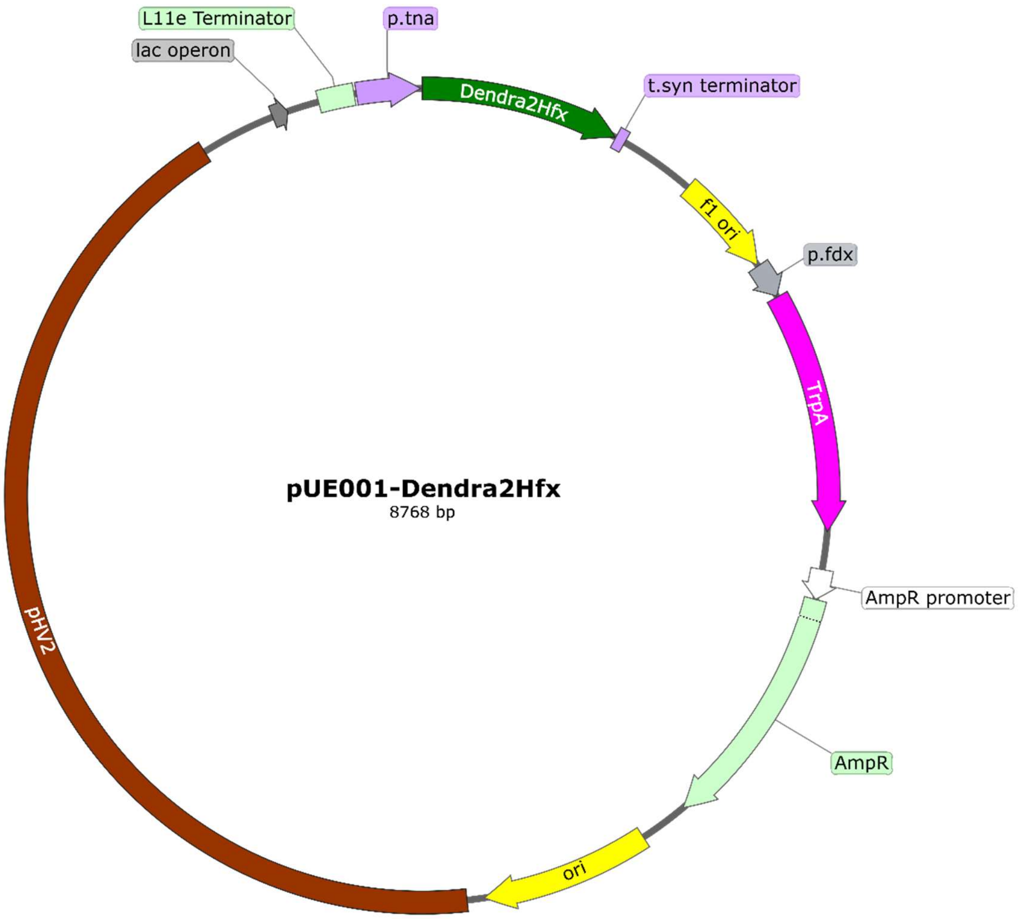

Supplementary Figure S1. Plasmid map of the novel expression system pUE001-Dendra2Hfx. pUE001 can be used as a shuttle vector in *E. coli* and *H. volcanii* and combines the selection marker trpA with the tryptophan inducible promoter p.tna.

Features for replication, selection and expression in *E. coli*: F1 ori: origin of replication; AmpR promoter: promoter for ampicillin resistance gene; *ampR*: ampicillin resistance gene for selection

Features for replication, selection, and expression in *H. volcanii: trpA*: tryptophan synthase encoding selection marker; p.fdx: constitutive promoter for the transcription of *trpA;* pHV2: origin of replication; p.tna: tryptophan inducible promoter for gene expression; L11e Terminator: rRNA terminator; t.syn: transcriptional terminator

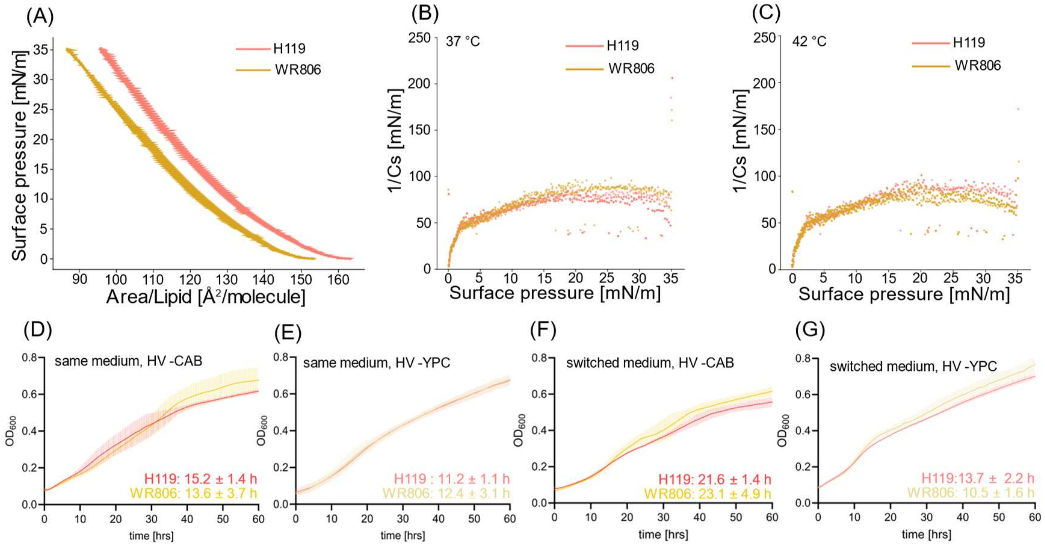

Supplementary Figure S2. Extended data for Figure 1 comparing strains H119 (red) and WR806 (yellow). (A) Area isotherms measuring surface pressure versus molecular area for lipid monolayers from extracted lipids from H119 and WR806 at 37 °C (biological duplicates, each with technical triplicates); (B, C) Compressibility modulus (k) of monolayer experiments performed with lipid extracts from H119 and WR806 at 37 °C (B) and 42 °C (C); (D – G) Growth curves and doubling times of H119 and WR806 in different media grown at 42 °C in a plate reader (means ± std. dev.; at least two biological replicates, each with two technical replicates). (D, E) Growth curves and doubling times of both strains grown in the same medium in pre- and main-cultures. (F, G) Doubling times of H119 and WR806 strains after transitioning from HV-YPC to HV-CAB media or vice versa. The indicated medium represents the final medium in which the strains were grown.

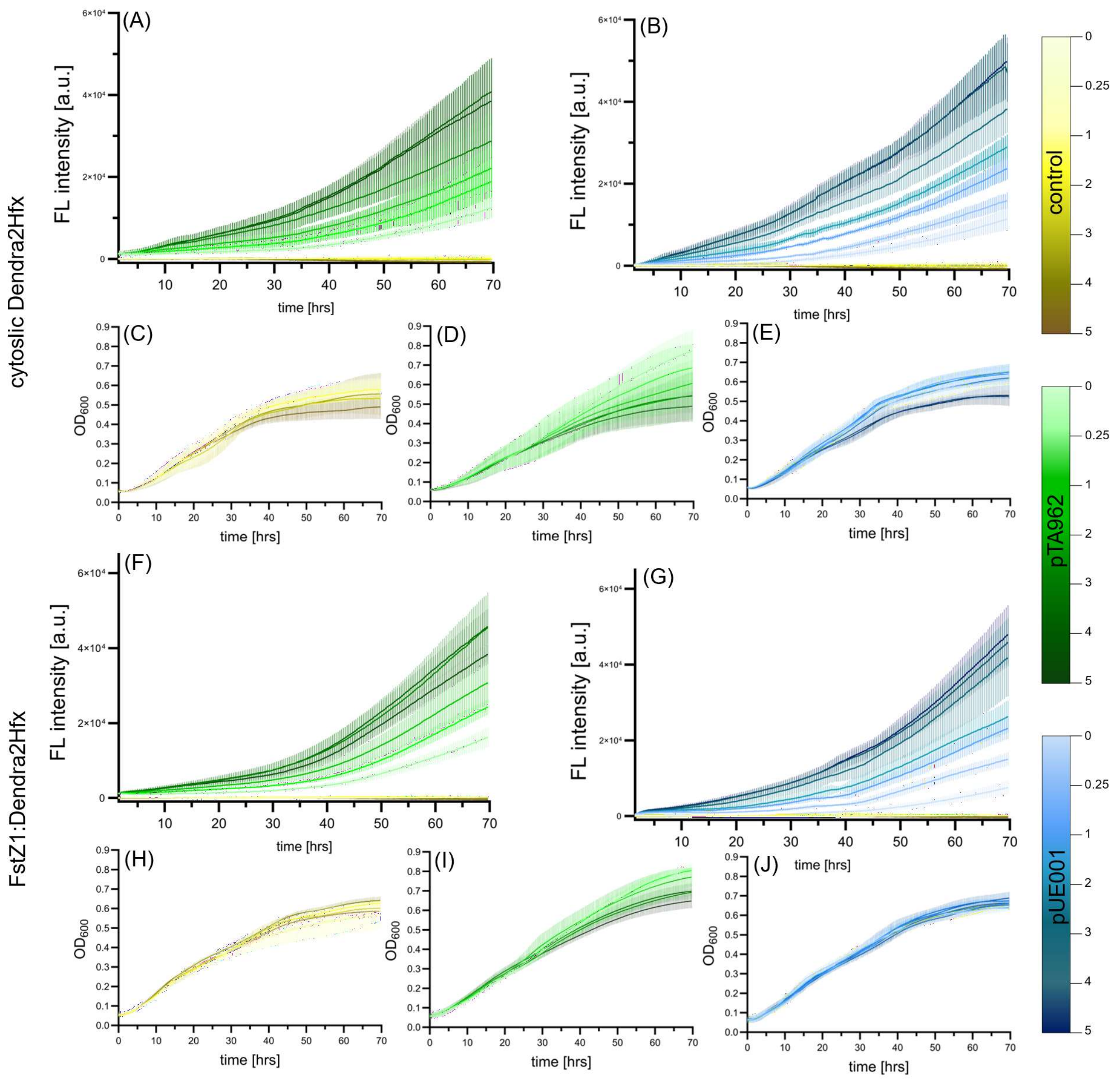

Supplementary Figure S3. Extended data to Figure 2. Fluorescence characterization of Dendra2Hfx expression in *H. volcanii* strain WR806. Raw fluorescence and OD measurements belonging to the normalized fluorescence intensities shown in Fig. 2A, B, D and E. Experiments were performed at 42°C in biological triplicates, each with technical duplicates, data is visualized as mean with standard deviation. Cultures carrying pTA962 (green) and pUE001 (blue) plasmids were induced with tryptophan concentrations of 0, 0.25, 1, 2, 3, 4, and 5 mM. Plasmid-free WR806 (yellow) served as negative control.

(A, B, F, G) Time-course fluorescence measurements of tryptophan-induced protein expression (ex 470 nm; em 501-519 nm, optimized for Dendra2Hfx green fluorescence). (A, B) Cytosolic Dendra2Hfx expression. (F, G) FtsZ1:Dendra2Hfx fusion protein expression.

(C, D, E, H, I, J) Measurement of optical density was determined by light absorption at a wavelength of 600 nm. (C, D, E) optical density measurements of WR806 (yellow), WR806 pTA962-Dendra2Hfx (green), and WR806 pUE001-Dendra2hfx (blue). (H, I, J) Optical density measurements of WR806 (yellow), WR806 pTA962-FtsZ1:Dendra2Hfx (green), and WR806 pUE001-FtsZ1:Dendra2hfx (blue).

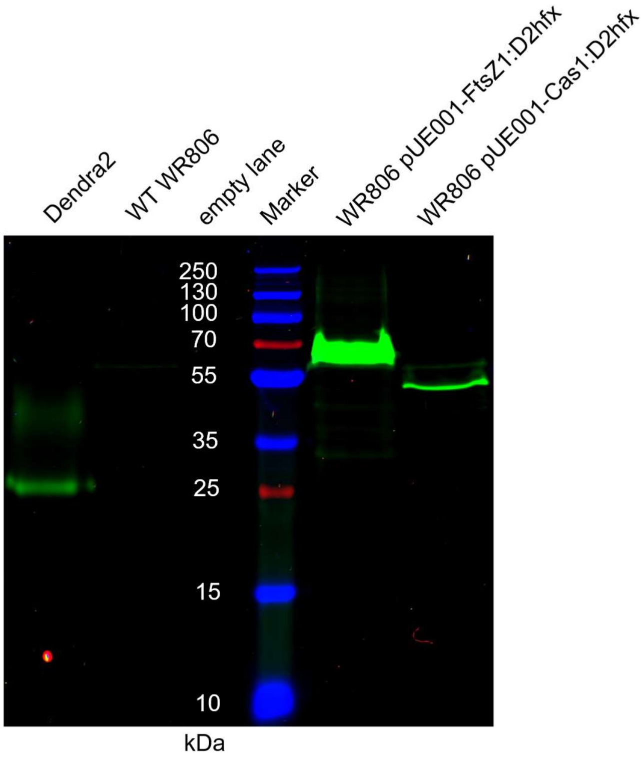

Supplementary Figure S4. SDS-PAGE with detected in-gel-fluorescence (Sanial et al., 2025) of purified Dendra2 (from *E. coli*), and cell lysates of WR806 wildtype, WR806 pUE001-FtsZ1:Dendra2Hfx, and WR806 pUE001-Cas1:Dendra2Hfx. The pUE001 carrying strains were induced with 4 mM tryptophan. Samples and marker (PageRuler Plus Prestained Protein Ladder (Thermofisher)) were run on a standard 12.5% discontinuous SDS-PAGE and visualized using the Cy2, 3 and 5 chanel of a Typhoon™ Amersham™ Biomolecular Imager (Cytiva, USA).

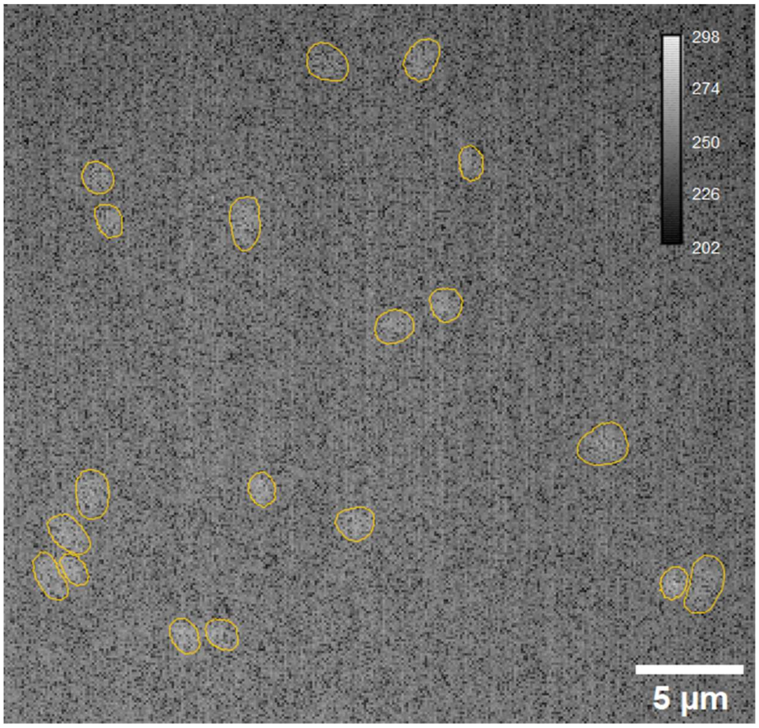

Supplementary Figure S5. The fluorescence of green Dendra2Hfx in WR806 pTA231-FtsZ1:Dendra2Hfx at OD 1 was measured. Although the strain carried the correct plasmid as verified by colony PCR, no visible expression of the fusion protein FtsZ1-Dendra2Hfx was detected. This finding is consistent with earlier, unpublished observations during tests of fusion constructs for plasmids described in (Turkowyd et al., 2020). While cytosolically expressed fluorescent proteins were clearly visible (Turkowyd et al., 2020), fusion proteins in a pTA231 background consistently failed to show expression, as observed in our unpublished work. We attribute this suppression to the potential strong regulation of constitutively expressed transcripts that include native *H. volcanii* gene sequences. Having carefully confirmed these old preliminary results with above findings, we are now confident in the validity of the negative results and thus to publish them.

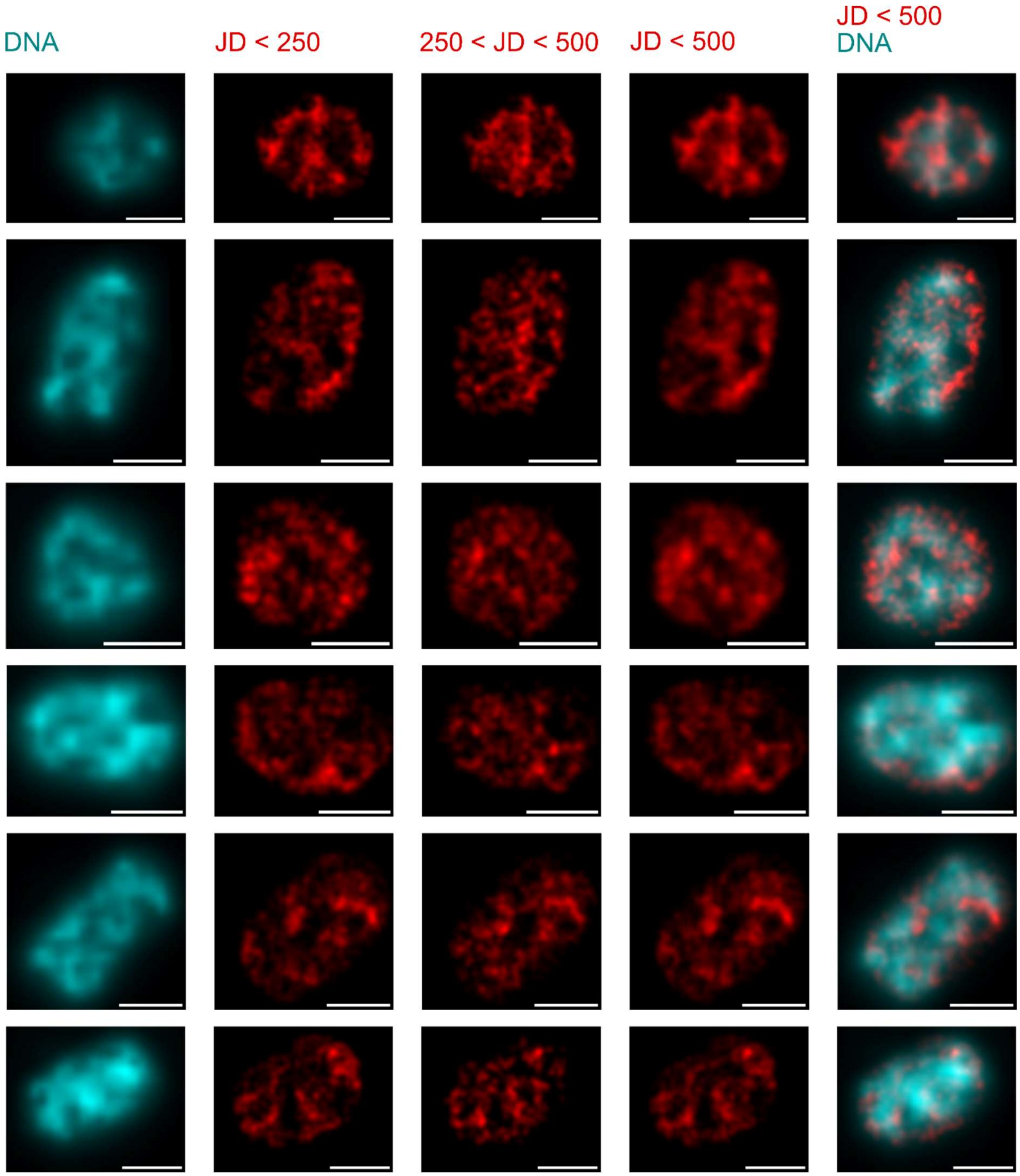

Supplementary Figure S6. Extended data to Figure 5 A**.** Exemplary images of WR806 pUE001-Cas1:Dendra2Hfx expressing cells that were stained with Hoechst 33342 to visualize DNA. DNA was imaged and deconvolved using Huygens Professional version 25.04 (Scientific Volume Imaging, The Netherlands, http://svi.nl) together with the setup-specific PSF. Cas1:Dendra2Hfx was measured using single-particle tracking microscopy. Trajectories of Cas1:Dendra2Hfx were tracked and localizations with jump distances (JD) under 250 nm, between 250 nm and 500 nm, and all trajectories under 500 nm were visualized using a gaussian blur filter (approximate localization precision of each localization 43 nm). Deconvolved DNA and visualized localizations with a JD under 500 nm were overlaid to a composite image. Scale bar 1 µm**.**

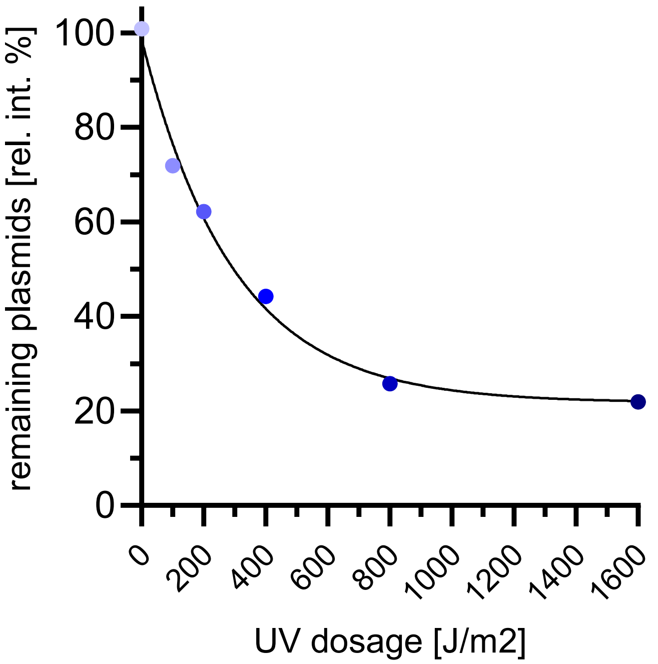

Supplementary Figure S7. Extended data to Figure 5 C. Exponential decay fit of the decrease of remaining plasmid DNA after Endo V digest upon increasing UV dosage. Dots indicate the fraction of remaining plasmid DNA. The values are identical with the values shown in Figure 5 C. Values of the 0 J/m^2 and 1600 J/m^2 condition are the mean. The black line indicates the fitted exponential decay function. The decay constant was fitted to $K = 3 \times 10^{-3}$ m^2/J.

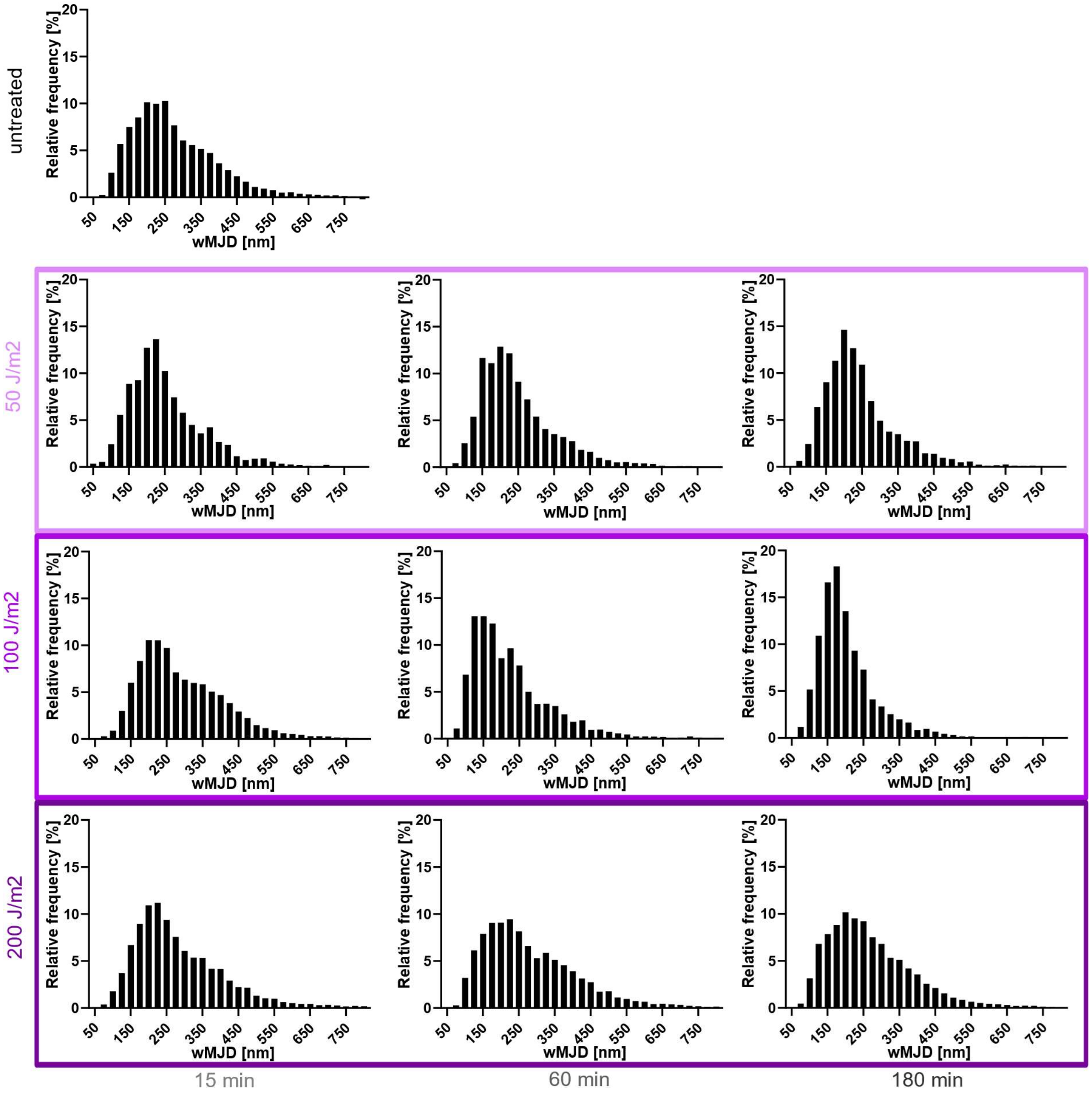

Supplementary Figure S8. Extended data to Figure 5D, E. Analysis of Cas1-Dendra2Hfx single-molecule dynamics in response to UV-light damage in *H. volcanii* strain WR806 pUE001 as measured by single-particle tracking microscopy. Exponentially growing cells cultured without tryptophan were sub-cultured with 0.25 mM tryptophan for inducing Cas1 protein expression overnight. Cells were reinoculated under the same conditions to OD 0.1 and after 2 hours of growth, exposed to UV-light radiation at 265 nm at 0, 50, 100, or 200 J/m² and measured after a recovery time of 0, 15, 60, and 180 minutes post-exposure. Dynamics are quantified by weighted mean jump distances (wMJD) from individual single-molecule trajectories across two biological replicates. Mean jump distances were weighted by the respective number of localizations per trajectory. Light to dark purple boxes indicate the increased UV dosage and histograms are sorted according to the recovery time of the cells after exposure. The untreated control is shown in the upper row.

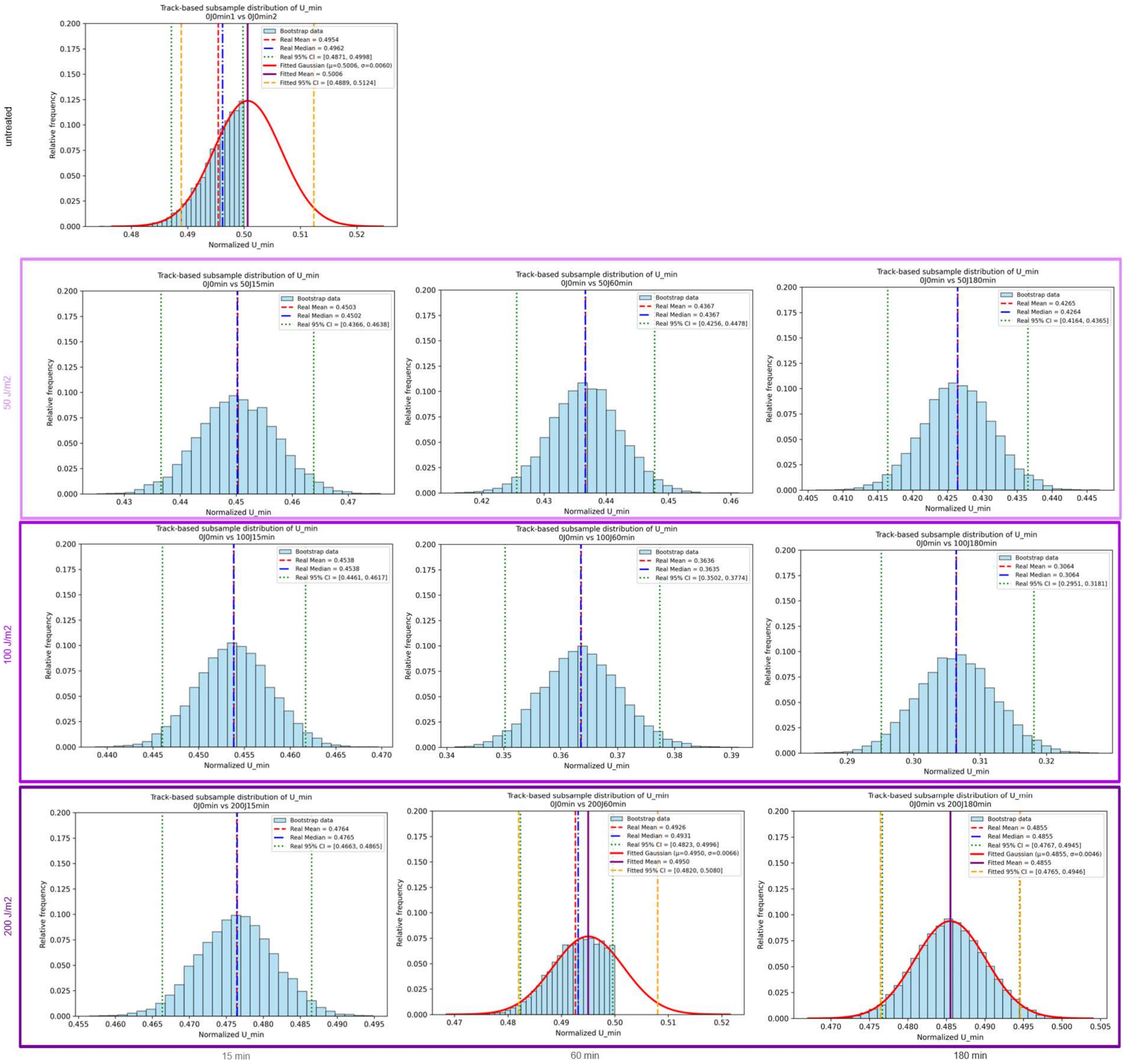

Supplementary Figure S9. Extended data to Figure 5E. Distributions of normalized U-values obtained by comparison of subsampled data sets of untreated wMJD and the indicated UV-treated wMJD data sets (raw data sets see Figure S7). Subsampling was performed by maintaining 60% of tracks in each data set and 10000 iterations, and mean median and 5% and 95% confidence intervals (CI) were determined. The untreated sample was split randomly into two halves, maintaining track information and compared to itself. As normalized U-values cannot exceed 0.5, a gaussian fit was applied to when histograms were cut off at 0.5 and the fitted mean and CI are plotted in Figure 5E. Blue bars represent the frequency distribution of subsampled data, dashed blue lines indicate the median of underlying data, dashed red lines indicate the mean of underlying data, Dashed green lines represent the 5% and 95% of subsampled data, continuous red lines indicated fitted gaussian distributions, continuous purple lines indicate the mean of fitted Gaussian distributions, Dashed orange lines represent the 5% and 95% of fitted Gaussian distributions.

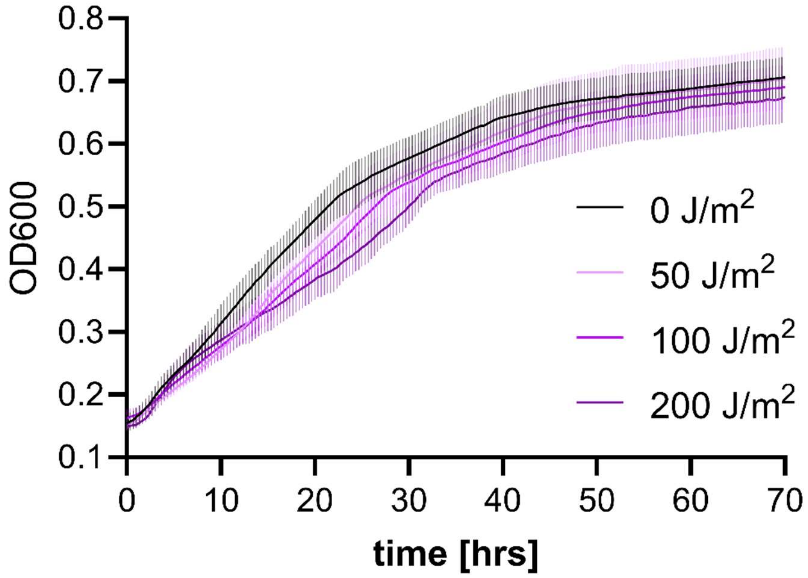

Supplementary Figure S10. Growth experiment of UV-treated WR806 pUE001-Cas1:Dendra2Hfx. The samples were treated with increasing UV-dosages immediately prior to starting the growth experiment. The growth experiments were conducted at 42 °C. Each sample was measured in two biological duplicates, each duplicate was measured in technical triplicates. The data is visualized as the mean with standard deviation.

References

Allers, T., Ngo, H. P., Mevarech, M., & Lloyd, R. G. (2004). Development of Additional Selectable Markers for the Halophilic Archaeon Haloferax volcanii Based on the leuB and trpA Genes. *Applied and Environmental Microbiology*, *70*(2). https://doi.org/10.1128/AEM.70.2.943-953.2004

Babu, M., Beloglazova, N., Flick, R., Graham, C., Skarina, T., Nocek, B., Gagarinova, A., Pogoutse, O., Brown, G., Binkowski, A., Phanse, S., Joachimiak, A., Koonin, E. V., Savchenko, A., Emili, A., Greenblatt, J., Edwards, A. M., & Yakunin, A. F. (2011). A dual function of the CRISPR-Cas system in bacterial antivirus immunity and DNA repair. *Molecular Microbiology*, *79*(2). https://doi.org/10.1111/j.1365-2958.2010.07465.x

McCready, S. (1996). The repair of ultraviolet light-induced DNA damage in the halophilic archaebacteria, Halobacterium cutirubrum, Halobacterium halobium and Haloferax volcanii. In *Mutation Research* (Vol. 364). ELSEVIER. https://doi.org/10.1016/0921-8777(96)00018-3

Rollie, C., Schneider, S., Brinkmann, A., Bolt, E., & White, M. (2015). Intrinsic sequence specificity of the Cas1 integrase directs new spacer acquisition. *ELIFE*. https://doi.org/10.7554/eLife.08716.001

Sanial, M., Miled, R., Alves, M., Claret, S., Joly, N., Proux-Gillardeaux, V., Plessis, A., & Léon, S. (2025). Direct observation of fluorescent proteins in gels: A rapid, cost-efficient, and quantitative alternative to immunoblotting. *Biology of the Cell*, *117*(2). https://doi.org/10.1111/boc.202400161

Stantial, N., Dumpe, J., Pietrosimone, K., Baltazar, F., & Crowley, D. J. (2016). Transcription-coupled repair of UV damage in the halophilic archaea. *DNA Repair*, *41*, 63–68. https://doi.org/10.1016/j.dnarep.2016.03.007

Turkowyd, B., Schreiber, S., Wörtz, J., Segal, E. S., Mevarech, M., Duggin, I. G., Marchfelder, A., & Endesfelder, U. (2020). Establishing Live-Cell Single-Molecule Localization Microscopy Imaging and Single-Particle Tracking in the Archaeon Haloferax volcanii. *Frontiers in Microbiology*, *11*. https://doi.org/10.3389/fmicb.2020.583010

Wörtz, J. (2022). *Haloferax volcanii: Untersuchung alternativer Funktionen der Endonuklease Cas1 über die CRISPR-Cas Immunabwehr hinaus*. Ulm University.

Wörtz, J., Smith, V., Fallmann, J., König, S., Thuraisingam, T., Walther, P., Urlaub, H., Stadler, P. F., Allers, T., Hille, F., & Marchfelder, A. (2022). Cas1 and Fen1 Display Equivalent Functions During Archaeal DNA Repair. *Frontiers in Microbiology*, *13*. https://doi.org/10.3389/fmicb.2022.822304
